# Regulation of microtubule nucleation in glioblastoma cells by ARF GTPase-activating proteins GIT1 and GIT2 and protein kinase C

**DOI:** 10.1186/s12935-025-03740-y

**Published:** 2025-04-02

**Authors:** Vadym Sulimenko, Eduarda Dráberová, Vladimíra Sládková, Tetyana Sulimenko, Věra Vosecká, Omar Skalli, Pavel Dráber

**Affiliations:** 1 Department of Biology of Cytoskeleton, Institute of Molecular Genetics, Czech Academy of Sciences, 142 20 Prague 4, Czech Republic; 2 Department of Biological Sciences, The University of Memphis, 101 Life Science Building, Memphis, TN 38152 USA

**Keywords:** Glioblastoma cells, Centrosomes, G protein-coupled receptor kinase-interacting proteins (GITs), Microtubule nucleation, Protein kinase C (PKC)

## Abstract

**Background:**

G protein-coupled receptor kinase-interacting proteins (GITs) function as GTPase-activating proteins (GAPs) for small GTPases of the ADP-ribosylation factor (Arf) family. While GIT proteins (GIT1 and GIT2) regulate both cell migration and microtubule organization, their corresponding regulatory mechanisms in glioblastoma cells remain largely unknown. To further investigate their role in microtubule modulation, we examined the function of GITs in microtubule nucleation and the involvement of protein kinase C (PKC) in this process.

**Methods:**

Glioblastoma cell lines with depleted GIT protein levels were generated using shRNA lentiviral vectors. The cellular localization of GITs was visualized by immunofluorescence microscopy, microtubule nucleation was analyzed using time-lapse imaging, and cell migration was assessed through a wound healing assay. Phosphomimetic and non-phosphorylatable variants of GIT2 were prepared by site-directed mutagenesis. Immunoprecipitation, pull-down experiments, and kinase assays in the presence of PKC inhibitors were used to study protein interactions.

**Results:**

Both GIT1 and GIT2 associate with proteins of the γ-tubulin ring complexes (γTuRCs), the primary microtubule nucleators, and localize to centrosomes. Depletion of GIT2 enhances centrosomal microtubule nucleation and has a more pronounced, yet opposite, effect on this process compared to GIT1. In contrast, the depletion of both GIT1 and GIT2 similarly affects cell migration. The N-terminal ArfGAP domain of GIT2 associates with centrosomes, regulates microtubule nucleation, and is phosphorylated by PKC, which modulates this process. We identified serine 46 (S46) on the ArfGAP domain as a PKC phosphorylation site and demonstrated that phosphorylation of GIT2 at S46 promotes microtubule nucleation.

**Conclusions:**

We propose that GIT2 phosphorylation provides a novel regulatory mechanism for microtubule nucleation in glioblastoma cells, contributing to their invasive properties.

**Supplementary Information:**

The online version contains supplementary material available at 10.1186/s12935-025-03740-y.

## Background

Gliomas are the most common group of central nervous system neoplasms, making up more than 70% of all brain tumors [1]. Among them, glioblastomas (isocitrate dehydrogenase wilde-type glioma, grade IV astrocytoma) are highly malignant brain tumors characterized by a heterogeneous population of genetically unstable and highly infiltrative cells [2]. Despite advances in our understanding of tumor biology and the development of new treatment strategies over the past decade, glioblastomas remain incurable, with a poor prognosis and median survival of 15 months after diagnosis [3]. Their aggressive invasiveness is associated with the aberrant expression of cytoskeletal proteins, including tubulins [4], intermediate-filament proteins [5], and actin-regulatory proteins [6]. To develop novel therapeutic approaches targeting cytoskeletal proteins, it is important to deepen our understanding of microtubule regulation in glioblastoma cells.

Microtubules, composed of α- and β-tubulin heterodimers, are highly dynamic cytoskeletal structures, which during interphase are primarily nucleated at the centrosome and extend toward the cell periphery. γ-Tubulin, a conserved but less abundant member of the tubulin superfamily, is essential for microtubule nucleation [7]. While multiple genes encode α- and β-tubulins in humans, only two genes [8] encode nucleation-competent γ tubulins [9]. Excessive and ectopic accumulation of γ-tubulin in primary gliomas has been linked to the acquisition of a more malignant phenotype [4]. Microtubule nucleation is driven by γ-tubulin ring complexes (γTuRCs), the primary microtubule nucleators, which act as templates for αβ-tubulin dimers to assemble into growing microtubules. The multi-subunit γTuRCs are composed of γ-tubulin and a series of γ-tubulin complex proteins (GCPs) 2–6 [10], along with other components such as actin and MZT 1/2 [11]. Proteins responsible for recruiting and activating γTuRCs at the centrosomes are essential for the spatiotemporal regulation of microtubule nucleation [12, 13]. Additionally, other proteins that indirectly influence microtubule nucleation play a significant role in this process. The activity of regulatory proteins modulating centrosomal microtubule nucleation can be finely controlled by phosphorylation [14].

G protein-coupled receptor kinase-interacting proteins (GITs) function as GTPase-activating proteins (GAPs) for small GTPases of the ADP-ribosylation factor (Arf) family [15]. GITs, which are evolutionary conserved and composed of multiple domains, act as adaptor proteins connecting Arf GTPases to other intracellular signaling pathways [16, 17]. There are two GIT proteins in humans: GIT1 and GIT2. While GIT1 has two splice variants of similar size, GIT2 undergoes more complex splicing, resulting in a full-length version (GIT2-long), and a shorter version (GIT2-short) that lacks the C-terminal domain [16]. GIT1 and GIT2-long (hereafter referred to as GIT2) share a similar domain architecture, featuring an N-terminal ArfGAP domain, three ankyrin repeat sequences, an SHD (Spa2-homology) domain, a coiled-coil domain, and a focal adhesion targeting (FAT) domain [17, 18]. GIT proteins contain multiple phosphorylation sites, though the specific kinases targeting these sites remain largely unknown [19].

GITs are involved in regulating cell adhesion and migration through their interaction with the actin cytoskeleton. They bind to p21-activated kinase interacting exchange factors (PIXs), which act as guanine nucleotide exchange factors (GEFs) for Rac/Cdc42 GTPases, modulating microfilament organization [18]. It has been also shown that GIT1 interacts with centrosomes, facilitating the activation of PAK1 kinase through a mechanism that does not involve GTPase activity [20]. Although GIT1 and GIT2 share similar domain structures, they may have distinct functions within the cell [17]. In previous studies, we found that both GIT1 and GIT2 associate with γ-tubulin in mouse bone-marrow derived mast cells, but they differently affect microtubule nucleation. GIT1 acts as a positive regulator [21], whereas GIT2 functions as a negative regulator [22]. Whether GIT1 and GIT2 also exhibit regulatory effects on microtubule nucleation in glioblastoma cells with elevated levels of γ-tubulin, both at the transcript and protein levels [4, 23], remains unclear.

In this study, we show that the depletion of GIT2 in glioblastoma cells has a more pronounced and opposite effect on centrosomal microtubule nucleation compared to GIT1. In contrast, the depletion of both GIT1 and GIT2 similarly affects cell migration. We also demonstrate that the ArfGAP domain of GIT2 and protein kinase C (PKC) are important for the regulation of microtubule nucleation. We identified the S46 site on the ArfGAP domain as a target for PKC and demonstrated that phosphorylation of GIT2 at S46 promotes microtubule nucleation. These results suggest that GIT2 phosphorylation provides a novel regulatory mechanism for microtubule nucleation in glioblastoma cells.

## Methods and materials

### Reagents

Calyculin A, Cytochalasin B, DMSO, DNAse I, Ficoll 400, G418 (Geneticin), Gö6983, nocodazole, phorbol 12-myristate 13-acetate (PMA), puromycin, and sotrastaurin were all sourced from Sigma-Aldrich (St. Louis, MO, USA). Hygromycin B was obtained from Invitrogen (Carlsbad, CA, USA), while polyethylenimine was purchased from Polysciences, Inc. (Warrington, PA, USA). Active human PKCα, tagged with GST and exhibiting a specific activity 3,200 nmol/min/mg, was acquired from Abcam (Cambridge, UK), whereas GST-tagged active human PAK1 (specific activity 63–85 nmol/min/mg) was supplied by Sigma-Aldrich. Protease-inhibitor mixture tablets (Complete EDTA-free) were sourced from Roche Molecular Biochemicals (Mannheim, Germany). Gö6976 was obtained from Calbiochem (San Diego, CA, USA), and chemiluminescent reagents (SuperSignal WestPico) were purchased from Pierce (Rockford, IL, USA). Protein A Sepharose CL-4B and Glutathione Sepharose 4 Fast Flow were procured from GE Healthcare Life Sciences (Chicago, IL, USA). Restriction enzymes were supplied by New England Biolabs (Ipswich, MA, USA), and Merck Life Science (Prague, Czech Republic) synthesized the oligonucleotides. Stock solution (1 mM) of PMA, and stock solutions (10 mM) of calyculin A, Gö6976, sotrastaurin, and Gö6983 were prepared in DMSO.

### Antibodies

The catalog numbers for commercially available antibodies (Abs) are indicated in parentheses. The mouse monoclonal Abs (mAbs) TU-30 (IgG1) and TU-31 (IgG2b), which recognize the γ-tubulin epitopes in the 434–449 region, have been previously described [24, 25]. The mAb GTU-88 (IgG1; T6557), specific for the γ-tubulin region 38–53, was sourced from Sigma-Aldrich. The mAbs to nucleolin (IgG1; sc-56640) and GCP4 (IgG1; sc-271876), as well as rabbit Abs targeting UBF1 (sc-9131) and GIT1 (sc-13961), were obtained from Santa Cruz Biotechnology (Dallas, TX, USA). The mAb TUB2.1 (IgG1, T4026), which detects β-tubulin, along with rabbit Abs specific for GAPDH (G9545), actin (A2066), and vinculin (V4505) were procured from Sigma-Aldrich. Rabbit Abs recognizing ODF2 (ab43840), and pericentrin for immunofluorescence applications (ab4448) originated from Abcam (Cambridge, UK). Rabbit Abs directed against calcineurin (2614) and P-Ser in PKC substrate motif (6967) were acquired from Cell Signaling Technology (Danvers, MA, USA). Rabbit Ab recognizing GIT2 (GTX133285) was sourced from Genetex (Irvine, CA, USA), while rabbit Ab against PKCα (21991-1-AP) came from Proteintech (Rosemont, IL, USA). Additionally, rabbit Ab specific for pericentrin for immunoblotting (ABT59) was obtained from Millipore (Temecula, CA, USA), and rabbit Ab to mNeonGreen (NG) (29523-1-AP) was provided by Proteintech. The mAb GCP2-02 (IgG1), which detects GCP2, has been previously characterized [26], as has the mAb GST-01 (IgG2b), which targets GST [22]. For negative control experiments in immunoprecipitation assays, a rabbit Ab against non-muscle myosin heavy chain (BT-561; Biomedical Technologies., Stoughton, MA, USA) and the mAb MT-03 (IgG2b), which binds the microtubule-associated protein MAP2ab [27], were used.

Secondary anti-rabbit (W401B), and anti-mouse (W402B) Abs conjugated with horseradish peroxidase (HRP) were obtained from Promega Biotec (Madison, WI, USA). Additionally, anti-rabbit Ab labeled with AF488 (115–545-146), and anti-mouse Ab conjugated with DyLight 549 (115–505-146) were purchased from Jackson Immunoresearch Laboratories (West Grove, PA, USA).

### Tissues, cell cultures, and transfection

A cadaveric human brain tissue sample for reverse transcription PCR was collected during an autopsy from a donor who died from a cause unrelated to brain disease. The sampling and storage procedures were previously outlined [25].

Human glioblastoma cell lines U-251 MG, U-373 MG, U-118 MG, and U-87 MG were acquired from the American Type Culture Collection (Manassas, VA, USA), while HEK293-FT (HEK) human kidney embryonal cells were bought from Promega Biotec. Cells were propagated in DMEM supplemented with 10% fetal bovine serum, 100 units/ml penicillin, and 0.1 mg/ml streptomycin. Cultures were maintained under standard conditions of 37 °C with 5% CO_2_ and passaged every 2 or 3 days using 0.25% trypsin and 0.01% EDTA in PBS (pH 7.5). Mycoplasma testing confirmed that cell lines were free from contamination. In addition, normal human fetal astrocytes (NHA), which are non-immortalized and non-transformed, were obtained from Cambrex Bio Science (Walkersville, MD, USA).

For transfection, glioblastoma cells were cultured in 24-well plates and transfected with 500 ng DNA per well, utilizing Lipofectamine 3000 in Opti MEM medium, following the manufacturer’s instructions (Invitrogen). After 5 h, the transfection medium was replaced with a fresh complete culture medium, and cells were incubated for an additional 48 h. HEK cells were transfected using polyethylenimine (Polysciences, Warrington, PA, USA) according to an established protocol [21].

For inhibition of Ser/Thr phosphatases, cells were exposed to 80 nM calyculin A for 30 min. PKC inhibition was achieved by preincubating cells for 30 min with either Gö6976 (10 μM), Gö6983 (10 μM), or sotrastaurin (10 μM). To activate PKCs, cells were treated with 1 μM PMA for 30 min.

### DNA constructs

To generate C-terminally mNeonGreen (NG)-tagged human GIT1, transcript variant 1 (tv1) (hGIT1, gene GIT1; RefSeq ID: NM_001085454), the coding sequence was amplified from pCR-hGIT1 [28] using primers containing recognition sites (underlined): forward 5'- ATTAGAATTCATGTCCCGAAAGGGGCC −3' and reverse 5'- ATTGTCGACAGCTGCTTCTTCTCTCGGG −3'. The PCR product was then digested and ligated into the pmNeonGreen-CT vector (Allele Biotechnology, San Diego, CA, USA), resulting in the plasmid phGIT1-NG. To prepare C-terminally NG-tagged human GIT2, transcript variant 1 (tv1) (hGIT2, gene GIT2; RefSeq ID: NM_057169), the coding sequence was amplified from hGIT2-Myc-DDK (OriGene Technologies, Rockville, MD, USA; RC212976) using primers with *NheI/SalI* recognition sites: forward 5'- ATTGCTAGCATGTCGAAACGGCTCCG −3' and reverse 5'- ACTGTCGACGCGTTGTTGTTCTCTTTGGT −3'. The PCR product was digested and ligated into the pmNeonGreen-CT vector (Allele), resulting in the plasmid phGIT2-NG. To create a C-terminally NG-tagged 1–124 amino acid (aa) region of human GIT2, the coding sequence was amplified from hGIT2-Myc-DDK (Origene) using primers with *NheI/SalI* recognition sites: forward 5′- ATTGCTAGCATGTCGAAACGGCTCCGG −3′ and reverse 5′- ATTGTCGACATATCCCGGCAGGGCAA −3′. The PCR product was digested and ligated into the pmNeonGreen-CT vector (Allele), generating the plasmid phGIT2_(1–124)_-NG. For the C-terminally NG-tagged 125–759 aa region of human GIT2, the coding sequence was amplified from hGIT2-Myc-DDK (Origene, RC212976) using primers with *NheI/SalI* recognition sites: forward 5′- CTGGCTAGCATGGACGATAGTGTGACTGC −3′ and reverse 5′- CTGGTCGACGCGTTGTTGTTCTCTTTG-3′. The PCR product was digested and ligated into the pmNeonGreen-CT vector (Allele), yielding the plasmid phGIT2_(125–759)_-NG.

To generate N-terminally GST-tagged full-length human GIT2(tv1), the coding sequence was amplified from the hGIT2-Myc-DDK (Origene) using primers with *SalI/NotI* recognition sites: forward 5′- ATTGTCGACATATGTCGAAACGGCTCCGGA −3′ and reverse 5′- ATGCGGCCGCTCAGTTGTTGTTCTCTTTG −3′. The PCR product was then digested and ligated into the pGEX-6P-1 vector (Amersham Biosciences, Uppsala, Sweden), resulting in the plasmid pGST-hGIT2_(1–759)_. To generate a GST-tagged 1–124 aa region of human GIT2, the coding sequence was amplified from hGIT2-Myc-DDK (Origene) using primers with *SalI/NotI* recognition sites: forward 5′- ATTGTCGACATATGTCGAAACGGCTCCGGA −3′ and reverse 5′- AAGCGGCCGCTCAATCCCGGCAGG −3′. The PCR product was digested and ligated into the pGEX-6P-1 vector (Amersham), resulting in the plasmid pGST-hGIT2_(1–124)_. For the GST-tagged 1–62 aa region of human GIT2, the coding sequence was amplified from hGIT2-Myc-DDK (Origene) using primers with *EcoRI*/*SalI* recognition sites: forward 5′- GCTCGAATTCATGTCGAAACGGCTCC −3′ and reverse 5′- ATCGTCGACTCACTGAAGCAGTGTTGGAG −3′. The PCR product was digested and ligated into the pGEX-6P-1 vector (Amersham), resulting in plasmid pGST-hGIT2_(1–62)_. For the GST-tagged 63–124 aa region of human GIT2, the coding sequence was amplified from hGIT2-Myc-DDK (Origene) using primers with *BamHI/SalI * recognition sites: forward 5′- GCCGGATCCATGGTTGAGACCTTGTAT −3′ and reverse 5 ‘- ATTGTCGACTCAATCCCGGCAGGG −3′. The PCR product was digested and ligated into the pGEX-6P-1 vector (Amersham), resulting in the plasmid pGST-hGIT2_(63–124)_. The human GIT2 aa 1–62 region was further divided into three parts by amplification from hGIT2-Myc-DDK (Origene) using primers with *EcoRI*/*SalI* recognition sites: forward 5 ‘- GCTCGAATTCATGTCGAAACGGCTCC −3 ‘ and reverse 5 ‘- ATTGTCGACTCAGGAAGGATCCGGC −3 ‘ for the 1–20 aa region; forward 5 ‘- GTCCGAATTCTGGGCATCAGTAAATAGGG −3 ‘ and reverse 5 ‘- ATTGTCGACTCAACTCCGATGGACACT −3 ‘ for the 21–40 aa region; forward 5 ‘- AGTCGAATTCCTAGGGCGCCATATCTCC −3 ‘ and reverse 5 ‘- ATCGTCGACTCACTGAAGCAGTGTTGGAG −3 ‘ for the 41–62 aa region. The PCR products were digested and ligated into the pGEX-6P-1 vector (Amersham), resulting in the plasmids pGST-hGIT2_(1–20)_, pGST-hGIT2_(21–40)_, and pGST-hGIT2_(41–62)_.

For phenotypic rescue experiments, three silent point mutations (c219t, t222a, a225t) were generated in the phGIT2-NG and phGIT2_(1–124)_-NG constructs by site-directed mutagenesis using the QuikChange II XL Site-Directed Mutagenesis Kit (Stratagene, La Jolla, CA), according to the manufacturer’s protocol. The resulting plasmids were named phGIT2-NGmut and phGIT2_(1–124)_-NGmut.

Plasmid pGST-hGIT1_(1–770)_, encoding N-terminally GST-tagged full-length human GIT1 was previously described [28], as was plasmid pGST-hTUBG1 encoding N-terminally GST-tagged full-length human γ-tubulin [29]. For the expression of NG-tagged EB3 and NG alone, plasmids pmNeonGreen-EB3-7 and pmNeonGreen-CT (Allele) were used. EB3-dtTomato was obtained from Addgene (Watertown, MA, USA; Cat. No 50708). The pLVX-mCherry-Actin lentiviral vector encoding human β-actin was obtained from Takara Bio (San Jose, CA, USA).

### RNA interference

Two short hairpin RNA (shRNA) constructs targeting human GIT1 (Gene ID: 28964; shRNA-GIT1-A, shRNA-GIT1-B) and two shRNA constructs targeting human GIT2 (Gene ID: 9815; shRNA-GIT2-A, shRNA-GIT2-B) were generated in the laboratory. The corresponding oligonucleotide sequences (Table S1) were selected using the Broad Institute's *GPP Web Portal* (https://portals.broadinstitute.org/gpp/public/). Sense and antisense oligonucleotides were annealed and inserted into the pLKO.1 hygro vector (Addgene) or the pLKO.1 puro vector (Addgene), both of which had been linearized with *Age*I*I*/*EcoRI*. The prepared vectors target all transcript variants of human GIT1 or GIT2. Immunoblotting experiments showed that the most efficient reduction of GIT1 and GIT2 protein levels was achieved with the vector shRNA-GIT1-A (GIT2_KD1) and the vector shRNA-GIT2-B (GIT2_KD2), respectively. As a negative control with hygromycin resistance, the pLKO.1 hygro vector, which contained annealed sense and antisense oligonucleotides (Table S1) derived from the pLKO.1-puro Non-Target shRNA Control Plasmid DNA (Sigma, Cat. No. SHC016), was employed. As a negative control with puromycin resistance, the pLKO.1-puro Non-Target shRNA Control Plasmid DNA was used.

### Lentiviral infection

Lentiviral infections were carried out as previously described [21], using HEK packaging cells for virus preparation. After 3 days, the transfection mixture was replaced with a fresh complete medium containing 1mg/ml hygromycin B. Stable selection was achieved by culturing the cells for 1–2 weeks. For the phenotypic rescue experiments, the medium containing hygromycin B was supplemented with Geneticin (1mg/ml), and stable selection was again achieved over 1–2 weeks. To prepare cells depleted of both GIT1 and GIT2, the stable GIT2-depleted cell line (GIT2_KD) with hygromycin resistance was infected with a lentiviral vector for GIT1 depletion with puromycin resistance. The stable selection was achieved by culturing the cells in a complete medium containing 2.5 µg/ml puromycin for 1–2 weeks.

THE pLVX-mCherry-Actin vector was transfected into HEK cells using the Lenti-X™ HT Packaging System (Takara Bio). Lentiviral particles were transduced into U373-MG cells as described [30], with 2 μg/ml puromycin selection applied for at least 8 days to ensure stable incorporation.

In phenotypic rescue experiments, GIT2_KD cells expressing GIT2-tagged proteins or NG alone were flow-sorted using a BD Influx cell sorter (BD Bioscience, San Jose, CA, USA). NG emission was triggered by a 488 nm laser; fluorescence was detected with a 530/30 band-pass filter.

### Site-directed mutagenesis

Phosphomimetic and non-phosphorylatable variants of GIT2 at position Ser 46 were generated in the phGIT2-NGmut and pGST-hGIT2_(1–62)_ plasmids. Phosphomimetic variants were prepared by replacing Ser 46 with Asp (S46D) using site-directed mutagenesis with the forward primer 5´- GTCTAGGGCGCCATATCGACCAAGTGAGGCATCTGAA −3´ and the reverse primer 5´- TTCAGATGCCTCACTTGGTCGATATGGCGCCCTAGAC −3´, generating phGIT2_(S46D)_-NG and pGST-hGIT2_(1–62)S46D_ plasmids. Non-phosphorylatable variants were prepared by replacing Ser 46 with Ala (S46A) using site-directed mutagenesis with the forward primer 5´- CTAGGGCGCCATATCGCCCAAGTGAGGCATC −3´ and the reverse primer 5´- GATGCCTCACTTGGGCGATATGGCGCCCTAG −3´, generating phGIT2_(S46A)_-NG and pGST-hGIT2_(1–62)S46A_ plasmids. The mutations were constructed using the QuikChange II XL Site-Directed Mutagenesis Kit (Stratagene) according to the manufacturer’s protocol.

### Reverse transcription PCR (RT-PCR)

Total RNA was extracted from the human glioblastoma cell line U-251 MG and cadaveric human brain tissue using the RNeasy Mini Kit (QIAGEN, Hilden, Germany) as previously described [25]. The RNA was then reverse-transcribed into cDNA using the SuperScript^®^ VILO cDNA Synthesis Kit with random primers (ThermoFisher Scientific, Waltham, MA, USA), according to the manufacturer’s instructions. PCR was performed using gene-specific primers for human PKCα (*PRKCA*, NCBI Ref. Seq.: NM_002737.3), PKCβ (*PRKCB*, NCBI Ref. Seq.: NM_212535.3, NM_002738.7; primers anneal to both transcript variants). PKCγ (*PRKCG*, NCBI Ref. Seq.: NM_001316329.2, NM_002739.5; primers anneal to both transcript variants), All primers were tested in silico using the Basic Local Alignment Search Tool from the National Center for Biotechnology Information (BLAST NCBI; NIH, Bethesda, MD, USA) to confirm specific amplification. Primer sequences are provided in Table S2. PCR was carried out as previously described [25], and the amplified fragments were separated on 2% agarose gels and stained by GelRed Nucleic Acid Gel Stain (Biotium, Fermont, CA, USA).

### Real-time qRT-PCR

Total RNA was isolated separately four times from glioblastoma cell lines (U-251 MG, U-118 MG, and U-87 MG) as well as from non-transformed normal human fetal astrocytes (NHA) utilizing the RNeasy Mini Kit (QIAGEN). The RNA was subsequently transcribed into cDNA with the High-Capacity cDNA Reverse Transcription Kit (Applied Biosystems, Waltham, MA, USA) employing random primers, following the manufacturer’s protocol. Quantitative PCR analysis was performed with specific primers targeting human γ-tubulin 1 (*TUBG1*, NCBI Ref. Seq.: NM_001070.5), human γ-tubulin 2 (*TUBG2*, NCBI Ref. Seq.: NM_001320509.2, NM_016437.3; primers cover both transcript variants), and β-actin (*ACTB*, NCBI Ref. Seq.: NM_001101.5). The specificity of all primers was confirmed through in silico BLAST analysis. Primer sequences are provided in Table S2. Quantitative PCR was conducted on a LightCycler 480 System (Roche Mannheim, Germany) as described [25], and the PCR products were validated by sequencing.

### Cell extracts

Whole-cell lysates for SDS-PAGE analysis were prepared by rinsing the cells with ice-cold HEPES buffer (50 mM HEPES, pH 7.6, 75 mM NaCl, 1 mM MgCl_2_, and 1 mM EGTA). The cells were then lysed in preheated SDS-sample buffer and subjected to boiling for 5 min. For immunoprecipitation and GST pull-down assays, cells cultured to approximately 90% confluence in 9-cm Petri dishes were rinsed with cold HEPES buffer before being extracted (1 ml per dish) on ice for 10 min at 4 °C using HEPES buffer supplemented with 1% NP-40 and both protease and phosphatase (1 mM NaF and 1 mM Na_3_VO_4_) inhibitors. The lysates were clarified by centrifugation at 20,000 × g for 15 min at 4 °C, and the resulting supernatant was collected for further analysis.

A previously established protocol [31] was adapted with modifications to analyze microtubule polymers. In brief, cells cultured in a six-well tissue plate were washed twice with pre-warmed PEM buffer (50 mM PIPES, pH 6.8, 3 mM MgCl_2_, and 1 mM EGTA) at 37°C. They were then extracted for 2 min at 37°C using 0.5 ml of PEM buffer containing 0.2% Triton X-100, 25% glycerol, and protease and phosphatase inhibitors. The resulting lysate was centrifuged at 1000 × g for 2 min at 25°C, allowing for the isolation of a nuclear pellet enriched in microtubules. The pellet was then resuspended in 0.4 ml of ice-cold lysis buffer (50 mM Tris, pH 7.4, 250 mM NaCl, and 5 mM EDTA) supplemented with 0.5% Triton X-100, and protease and phosphatase inhibitors. The mixture was incubated on ice for 5 min, and before immunoblotting, the samples were diluted 1:8 in SDS-sample buffer**.**

To obtain the nuclear fraction containing centrosomes, cells grown in a 6-cm Petri dish were detached with trypsin, rinsed twice with ice-cold HEPES buffer, and disrupted using a Dounce homogenizer in 1.2 ml of chilled HEPES buffer containing protease and phosphatase inhibitors. The efficiency of cell disruption was evaluated under a microscope. The homogenized mixture was then centrifuged at 300 × g for 5 min at 4 °C, resulting in the separation of a post-nuclear supernatant (S1) and a nuclear pellet (P1).

### Isolation of centrosomes

Centrosomes from U-251 MG cells were isolated using a modified version of a previously established protocol [32]. Briefly, cells were cultured in ten 15-cm Petri dishes to approximately 90% confluence. To induce microtubule and actin filament depolymerization, cells were treated with 10 μg/ml nocodazole and 5 μg/ml Cytochalasin B for 90 min at 37 °C. The cells were then rapidly sequentially washed with ice-cold PBS, followed by 8% (w/w) sucrose in 0.1 × PBS, 8% (w/w) sucrose in dd water, and a low-ionic-strength buffer (1mM Tris–Cl, pH 8.0, 8 mM 2-mercaptoethanol). Cells were lysed in low-ionic-strength buffer containing 0.5% NP-40 (9 ml per dish) for 20 min at 4 °C. The lysate was then centrifuged at 1,500 × g for 5 min at 4 °C to remove cellular debris. Centrosomes were enriched by centrifugation onto a 20% (w/w) Ficoll cushion at 25,000 × g, 20 min, 4 °C. Further purification was achieved through discontinuous sucrose gradient centrifugation as described [22]. For immunostaining, centrosomes were pelleted onto glass coverslips and fixed in methanol [33]. To evaluate their ability to nucleate microtubules, the isolated centrosomes were incubated with polymerization-competent 8 μM porcine brain tubulin in the presence of 1 mM GTP at 37℃ for 20 min as described [22].

### Immunoprecipitation, kinase assay, GST pull-down assay, gel electrophoresis, and immunoblotting

Immunoprecipitation was carried out following a previously established protocol [34]. Cell lysates were incubated with protein A beads preloaded with specific Abs. The mAbs used targeted γ-tubulin (TU-31; IgG2b) and MAP2 (MT-03; IgG2b) as a negative control. Rabbit Abs were used against GIT1, GIT2, PKCα, mNeonGreen (NG), and non-muscle myosin (another negative control). As an additional control, lysates were also incubated with protein A beads alone. The working concentrations of Abs for GIT1, GIT2, PKCα, and NG, were 0.4 μg/ml, 0.4 µg/ml, 1.2 μg/ml, and 1 μg/ml, respectively. The Ab to myosin was diluted 1:500. Hybridoma supernatants containing TU-31 and MT-03 mAbs were diluted 1:3 and 1:4, respectively.

The immunoprecipitated material bound to the beads was also used for in vitro kinase assays, following a previously established protocol [34]. The resulting ^32^P-labeled immunocomplexes were separated by SDS-PAGE, transferred onto membranes, and visualized by autoradiography using an Amersham Typhoon scanner (GE Healthcare Europe GmbH, Freiburg, Germany). The production and purification of soluble GST-tagged fusion proteins were performed according to an established protocol [35], and pull-down assays using whole-cell extracts were carried out as described [34]. The GST-tagged proteins used in the pull-down assays are shown in Fig. S1. To evaluate the phosphorylation of truncated GIT2 proteins, the immobilized GST-tagged proteins on beads were incubated with active PKCα (10 ng) in the presence or absence of 10 μM Gö6976 before kinase assays. Alternatively, kinase assays were conducted with GST-tagged proteins and active PAK1 (100 ng).

SDS-PAGE sample preparation, gel electrophoresis, and immunoblotting were performed following standard protocols [34, 36]. For immunoblotting, the mAb against γ-tubulin (Sigma, GTU-88) was diluted 1:10,000. The mAbs against β-tubulin, GCP4, and nucleolin were used at a 1:1,000 dilution. The mAbs against GST and GCP2, derived from hybridoma supernatants, were diluted 1:5,000, and 1:5, respectively. Rabbit Abs against GIT2, pericentrin (Millipore), ODF2, calcineurin, and UBF1 were diluted 1:1,000. Rabbit Abs specific to GAPDH, actin, mNeonGreen, GIT1, P-Ser in phospho-PKC motif, and PKCα were used at dilutions of 1:500,000, 1:30,000, 1:10,000, 1:5,000, 1:2,000, and 1:2,000, respectively. HRP-conjugated secondary Abs against mouse and rabbit IgG were diluted 1:10,000. Chemiluminescent detection was performed using SuperSignal WestPico reagents, and signals were visualized with the LAS 3000 imaging system (Fujifilm, Düsseldorf, Germany). Immunoblot signal quantification was carried out using AIDA image analyzer software (version 5; Raytest, Straubenhardt, Germany).

### Wound healing assay

To prepare defined cell-free areas for wound healing assay, we used a removable biocompatible insert in the cell monolayer as described [37]. A 70 μl suspension of U-251 MG cells (4 × 10^5^ cells) was applied to each well of the Culture-Insert 3 Well in μ-Dish 35 mm high ibiTreated (Ibidi GmbH, Gräfelfing, Germany; Cat. No. 80366), and a confluent layer was obtained after 24 h. The culture insert was removed, the cells were washed, and 2 ml of media with 2% serum was added to the dish. Images were captured at various time intervals (0–40 h) using an inverted wide-field microscope Olympus IX81 (Olympus, Hamburg, Germany). Image analysis was performed using the Image J plugin for the high throughput analysis [38].

### Microtubule nucleation visualized by time-lapse imaging

Glioblastoma cells expressing either EB3-mNeonGreen or EB3-dtTomato (used in phenotypic rescue experiments) were plated in a 35 mm µ-Dish with a polymer coverslip bottom (Ibidi). Thirty minutes before imaging, the culture medium was replaced with FluoroBrite™ DMEM containing 25 mM HEPES and 2% FCS. Live cell imaging was conducted at 1-s intervals over 30 s, capturing five optical slices taken at 0.25 µm increments. Imaging was performed using an Andor Dragonfly 503 spinning disc confocal system (Oxford Instruments, Abingdon, UK) equipped with a stage top incubator (Okolab, Ottaviano, Italy), an HCX PL APO 63x/1.4 oil immersion objective, and iXon Ultra 888 EMCCD camera. For EB3-mNeonGreen imaging, a 488 nm solid-state 150 mW laser with a 525/50 nm emission filter was utilized, while EB3-dtTomato imaging employed a 561 nm solid-state 100 mW laser with a 600/50 nm emission filter. At least 14 cells were imaged per experiment, with acquisition parameters set to a 40 µm pinhole size, 6% laser power, and a 70 ms exposure time. Maximum intensity projections of z-stacks were generated for each time point using the Fiji Processing program [39], and the time-lapse sequences were deconvoluted with Huygens Professional software v. 19.04 (Scientific Volume Imaging, Hilversum, the Netherlands). Newly nucleated microtubules were identified by manual tracking and counting EB3 comets originating from the centrosomes.

### Immunofluorescence microscopy

Immunofluorescence microscopy was carried out following established protocols [24]. Cells were extracted with 0.2% Triton X-100, followed by fixation in 3% formaldehyde and subsequent post-fixation in chilled methanol (Tx/F/M). Isolated centrosomes were fixed in methanol alone (M), whereas those involved in microtubule nucleation were fixed with glutaraldehyde and then post-fixed in methanol (G/M). For actin visualization, samples were fixed in formaldehyde (F). A mAb against γ-tubulin (TU-30), derived from spent culture supernatant, was used at a 1:10 dilution, while mAbs against β-tubulin and vinculin were diluted 1:500 and 1:100, respectively. A rabbit Ab targeting pericentrin was diluted 1:200. The DY549-conjugated anti-mouse Ab and the AF488-conjugated anti-rabbit Ab were applied at dilutions of 1:1,000, and 1:200, respectively. Samples were examined using an Olympus AX-70 Provis microscope (Olympus) equipped with a 60 × /1.0 NA water-immersion objective.

### Statistical analysis

For each quantification, data from at least three independent experiments were analyzed. The number of individual data points is specified in the figure legends. All values are expressed as mean ± SD. Statistical significance was assessed using either a two-tailed, unpaired Student’s *t*-test or one-way ANOVA followed by Dunnett’s or Sidak’s post hoc test, performed with Prism software v. 10.0.1 (GraphPad Software, San Diego, CA, USA). The specific statistical test applied is noted in the figure legends. *p* values were represented as follows: ns (p > 0.05), *** (*p* < 0.001), and **** (*p* < 0.0001).

## Results

### GIT1 and GIT2 associate with γTuRC proteins in glioblastoma cells

**Fig. 1:**
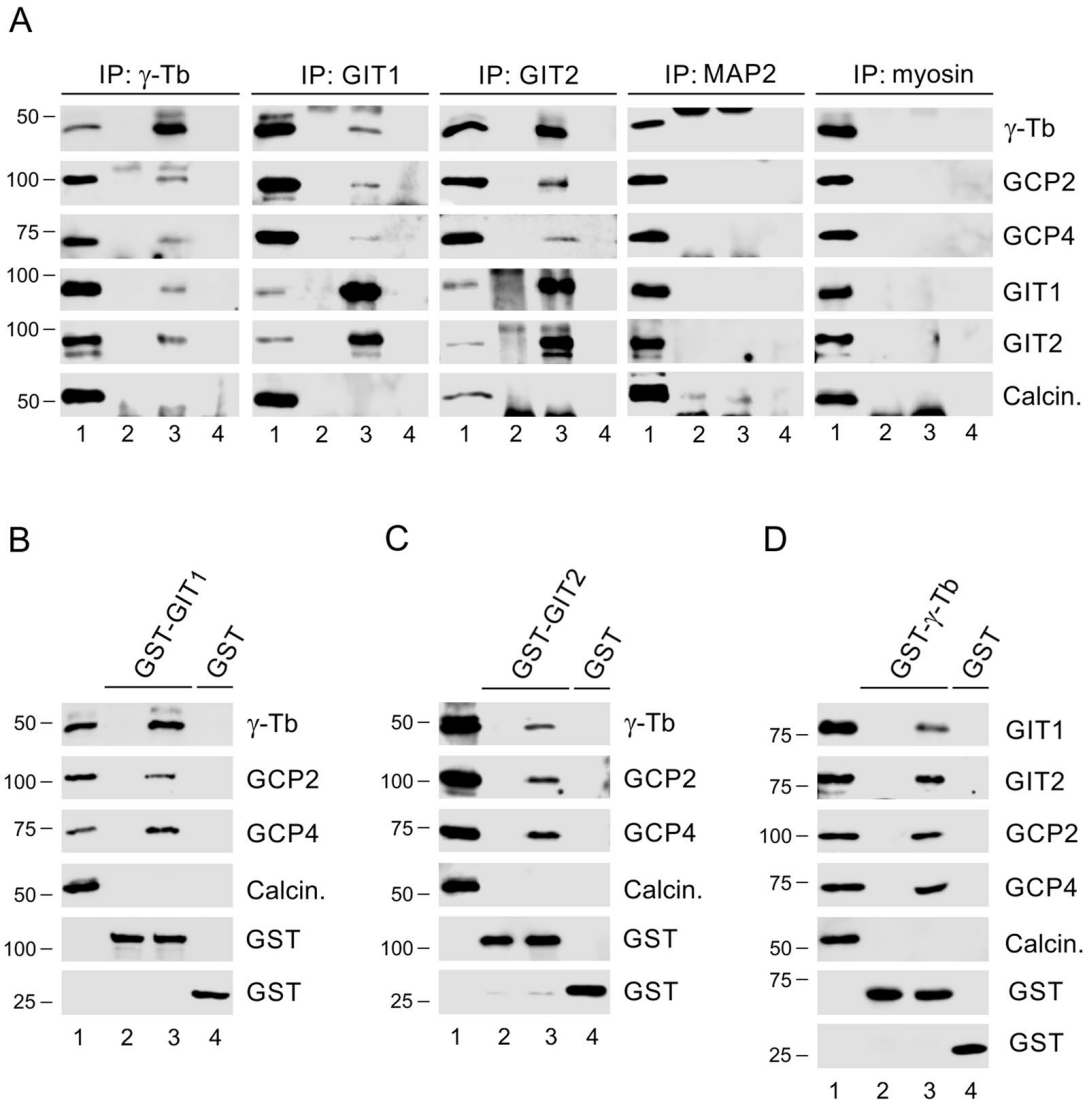
GIT1 and GIT2 interact with γTuRC proteins. Immunoprecipitation and GST pull-down experiments were performed using whole-cell extracts from U-251 MG cells. (**A**) Immunoprecipitation. Extracts were precipitated with immobilized rabbit Ab to GIT1, rabbit Ab to GIT2, mouse mAb TU-31 (IgG2b) to γ-tubulin (γ-Tb), rabbit Ab to myosin (isotype control), or mouse mAb to MAP2 (IgG2b; isotype control). The blots were probed with Abs to γ-tubulin (γ-Tb), GCP2, GCP4, GIT1, GIT2, or calcineurin (Calcin.; negative control). Load (*lane 1*), immobilized Abs without cell extracts (*lane 2*), precipitated proteins (*lane 3*), and Ab-free carriers incubated with cell extracts (*lane 4*). (**B**–**D**) GST pull-down assay. GST-fusion proteins or GST alone were immobilized on Glutathione-Sepharose and incubated with extracts. Pull-downs were conducted with GST-GIT1 (**B**), GST-GIT2 (**C**), and GST-γ-tubulin (GST-γ-Tb) (**D**). The blots were probed with Abs to γ-tubulin (γ-Tb), GCP2, GCP4, GIT1, GIT2, calcineurin (Calcin.; negative control), and GST. Load (*lane 1*), GST-fusion proteins without cell extracts (*lane 2*), proteins bound to GST-fusion proteins (*lane 3*), and proteins bound to GST alone (*lane 4*)

To determine whether GIT1 and GIT2 exhibit regulatory effects on microtubule nucleation in human glioblastoma cells, characterized by enhanced expression of both γ-tubulin isoforms (Fig. S2), we first conducted immunoprecipitation experiments using whole-cell extracts from U-251 MG glioblastoma cells. By employing mAb to γ-tubulin (IgG2b) and rabbit Abs to GIT1 and GIT2 for reciprocal immunoprecipitations, we revealed an association of both GIT1 and GIT2 with γ-tubulin, as well as with GCP2 and GCP4, which are components of the γTuRC. No association was observed with calcineurin, a Ser/Thr protein phosphatase used as a negative control. Additionally, mAb to microtubule-associated protein 2 (MAP2) (IgG2b) and rabbit anti-myosin Ab, used as isotype controls for the immunoprecipitation experiments, did not co-precipitate γ-Tb, GCP2, GCP4, GIT1 or GIT2 (Fig. 1A).

To independently confirm the interaction of GIT1 and GIT2 with γTuRC proteins, we performed immunoprecipitation experiments using cells expressing NG-tagged GIT1 and GIT2, as well as control cells expressing NG alone. The anti-NG Ab co-precipitated γ-tubulin, GCP2, and GCP4 from cells expressing GIT1-NG (Fig. S3A) or GIT2-NG (Fig. S3B), but did not co-precipitate these proteins from cells expressing NG alone (Fig. S3C). Reciprocal precipitation with the anti-γ-tubulin Ab further validated the interaction of γ-tubulin with GIT1-NG (Fig. S3D) or GIT2-NG (Fig. S3E).

Finally, we verified the interaction between GIT1 and GIT2 with γTuRC proteins by pull-down assays using GST-tagged GIT1, GIT2, and γ-tubulin. Both γ-tubulin, GCP2 and GCP4 demonstrated binding to GST-GIT1 (Fig. 1B) and GST-GIT2 (Fig. 1C), but not to GST alone. Conversely, GIT1 and GIT2 bound to GST-γ-tubulin (Fig. 1D).

These results collectively demonstrate that both endogenous and exogenous GIT1 and GIT2 associate with γTuRC proteins in U-251 MG glioblastoma cells.

### GIT1 and GIT2 associate with centrosomes in interphase and mitotic cells

**Fig. 2:**
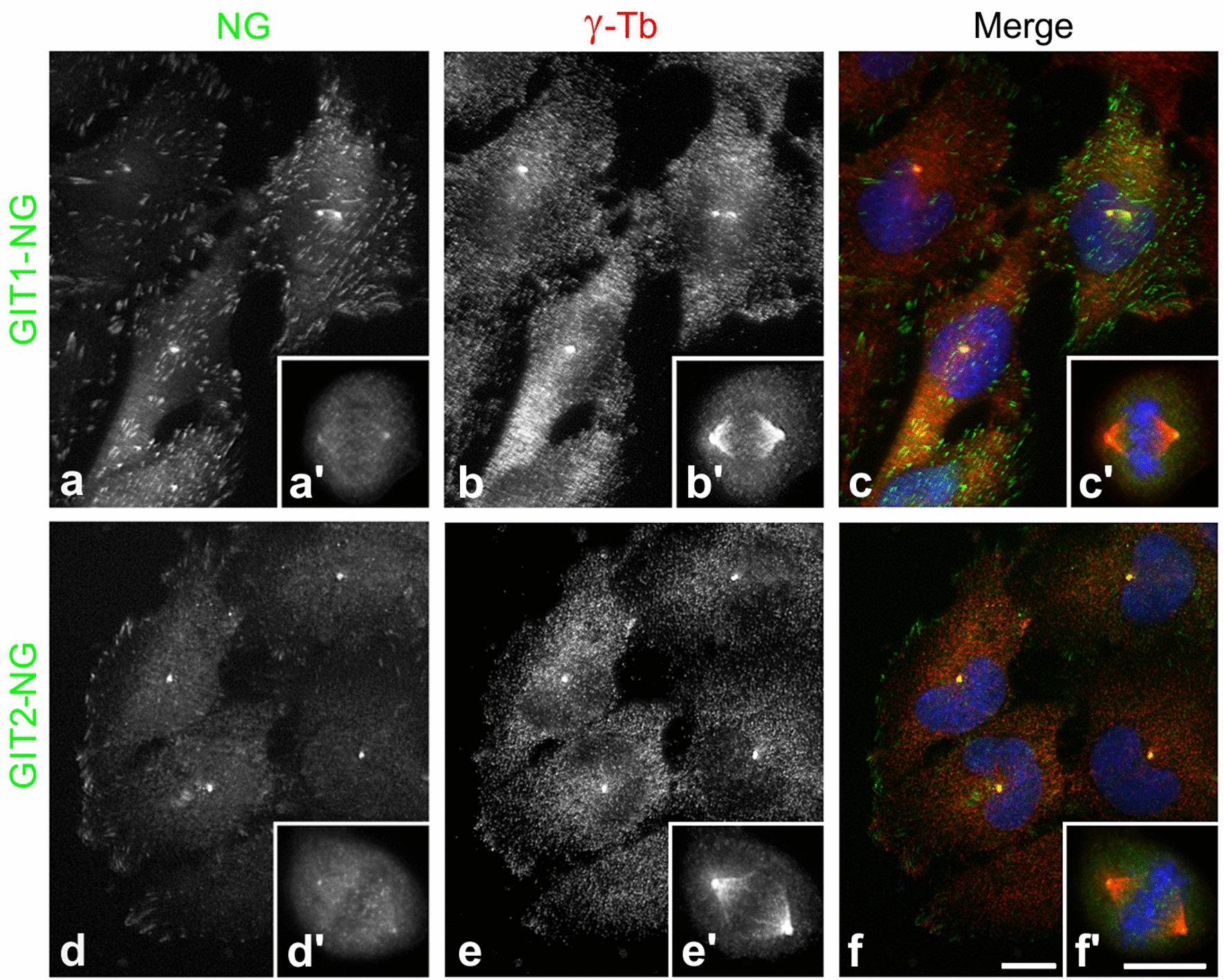
Subcellular localization of exogenous GIT1 and GIT2. U-251 MG cells expressing mNeonGreen-tagged GIT1 (GIT1-NG) or GIT2 (GIT2-NG) were fixed and stained with Ab to γ-tubulin. GIT1-NG (**a, a’**), γ-tubulin (**b, b’**; γ-Tb), and superposition of images (**c, c’**; GIT1-NG in green, γ-tubulin in red, DAPI in blue). GIT2-NG (**d, d’**), γ-tubulin (**e, e’**; γ-Tb), and superposition of images (**f, f’**; GIT2-NG in green, γ-tubulin in red, DAPI in blue). Cells in interphase are shown in panels **a-f**, while dividing cells in metaphase are shown in the insets **a’-f’**. The images (**a, d**; **a’ d’**; **b, e**; **b’, e’**) were collected and processed in the same manner. Fixation Tx/F/M. Scale bars, 10 μm (**a-f, a’-f’**)

Given the critical role of γTuRCs in centrosomal microtubule nucleation, we aimed to determine whether GIT1 and GIT2 localize to the centrosome. Initial attempts using immunofluorescence microscopy with a limited selection of commercially available antibodies against GIT1 and GIT2 did not reveal their presence at the centrosomes. Consequently, we expressed GIT1-NG, GIT2-NG, and NG alone (as a negative control) in U-251 MG cells. Both GIT1-NG and GIT2-NG were expressed at comparable levels, and their overexpression did not affect actin expression levels (Fig. S4A). In fixed U-251 MG cells expressing NG-tagged proteins, we marked centrosomes and focal adhesions using Abs to γ-tubulin and vinculin, respectively. GIT1-NG localized to the interphase centrosomes (Fig. 2a–c) and focal adhesions (Fig. S4B, a-c). GIT2-NG predominantly localized to centrosomes (Fig. 2d–f), with less obvious association with focal adhesions (Fig. S4B, d-f). Both tagged proteins also bound to spindle poles in metaphase, shown for GIT1-NG in Fig. 2a’–c’ and for GIT2 in Fig. 2 d’-f’. The differential subcellular localization of GIT1-NG and GIT2-NG was consistent in U-373 MG cells expressing mCherry-actin, which delineates the cell periphery. While GIT1 clearly associated with actin-positive focal adhesions (Fig. S4C, a-c), this was less apparent for GIT2-NG (Fig. S4C, d-f). In contrast, NG alone did not localize to centrosomes (Fig. S4C, g-i).

**Fig. 3:**
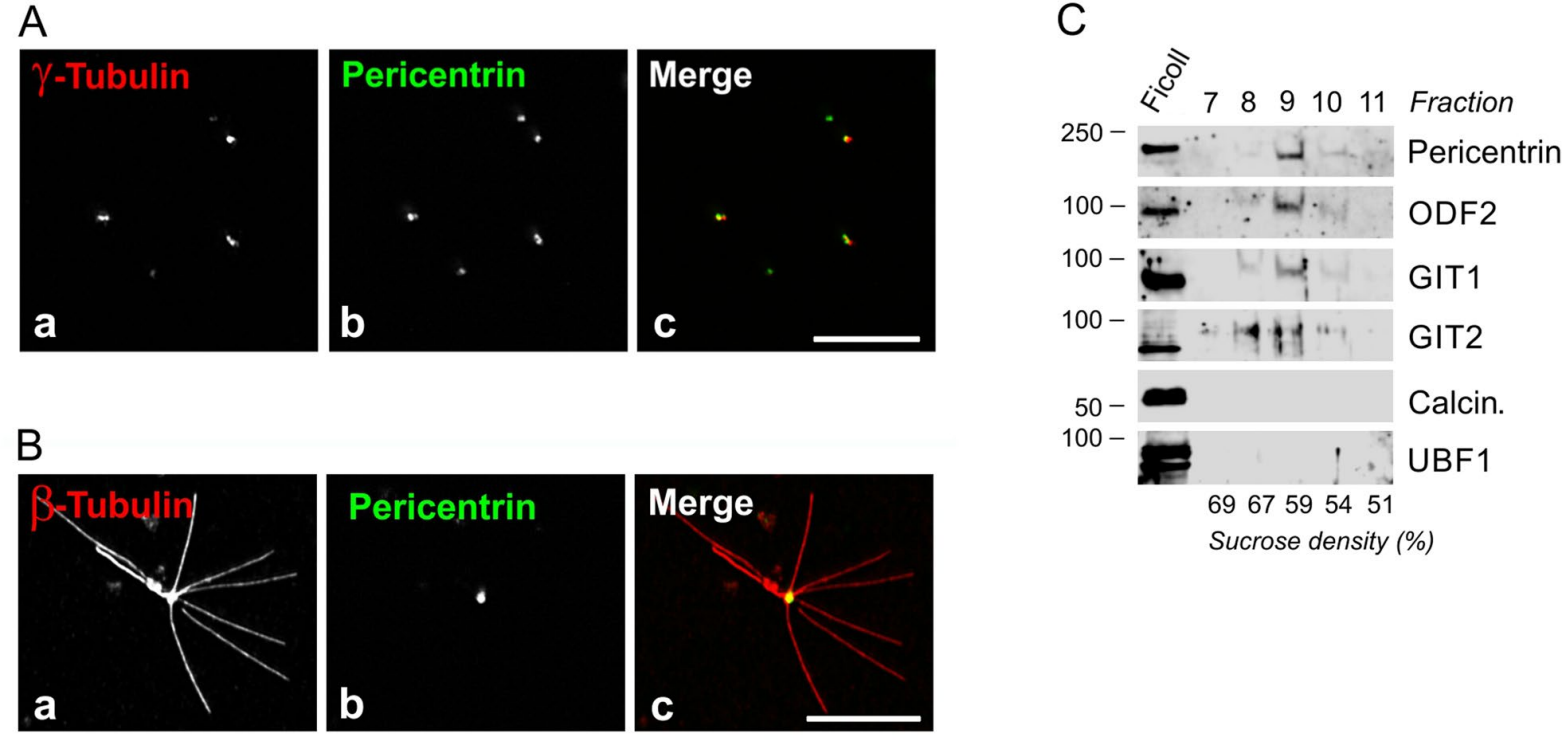
Association of endogenous GIT1 and GIT2 with purified centrosomes. Centrosomes were isolated from U-251 MG cells by sucrose gradient centrifugation. (**A**) Double-label staining of centrosomes pelleted on a coverslip with Abs to γ-tubulin and pericentrin. γ-Tubulin (**a**), pericentrin (**b**), and superposition of images (**c;** γ-tubulin in red and pericentrin in green). Fixation with methanol. Scale bar, 10 μm (**a-c**). (**B**) Functional assay of isolated centrosomes. Centrosomes were incubated in suspension with 8 μM tubulin in the presence of 1 mM GTP for 20 min at 37℃ and were thereafter fixed with glutaraldehyde, pelleted onto a coverslip, postfixed with methanol (Fixation G/M), and double-label stained with Abs to β-tubulin and pericentrin. β-Tubulin (**a**), pericentrin (**b**), superposition of images (**c**; β-tubulin in red and pericentrin in green). Scale bar, 10 μm (**a**–**c**). (**C**) Immunoblot of fractions from sucrose gradient centrifugation. The gradient was fractionated from the bottom, with individual fractions indicated at the top. Sucrose density in each fraction is shown at the bottom. Blots were probed with Abs to pericentrin, ODF2, GIT1, GIT2, calcineurin (Calcin.), and UBF1

To investigate whether GIT1 and GIT2 are associated with centrosomes, we isolated centrosomes from U-251 MG cells through gradient centrifugation. Immunofluorescence staining of purified centrosomes using Abs against γ-tubulin and pericentrin revealed distinct, brightly stained puncta (Fig. 3A). Furthermore, the isolated centrosomes retained their ability to nucleate microtubules when supplemented with 8 μM tubulin and 1 mM GTP (Fig. 3B). Immunoblot analysis detected GIT1 and GIT2 in the same fractions as pericentrin and outer dense fiber 2 (ODF2), both well-established centrosomal markers. The cytosolic and nuclear marker proteins, calcineurin and UBF1 were absent in these fractions (Fig. 3C).

These findings corroborate that both endogenous and exogenous GIT1 and GIT2 are associated with centrosomes in glioblastoma cells.

### GIT1 and GIT2 differentially regulate centrosomal microtubule nucleation but not cell migration

**Fig. 4:**
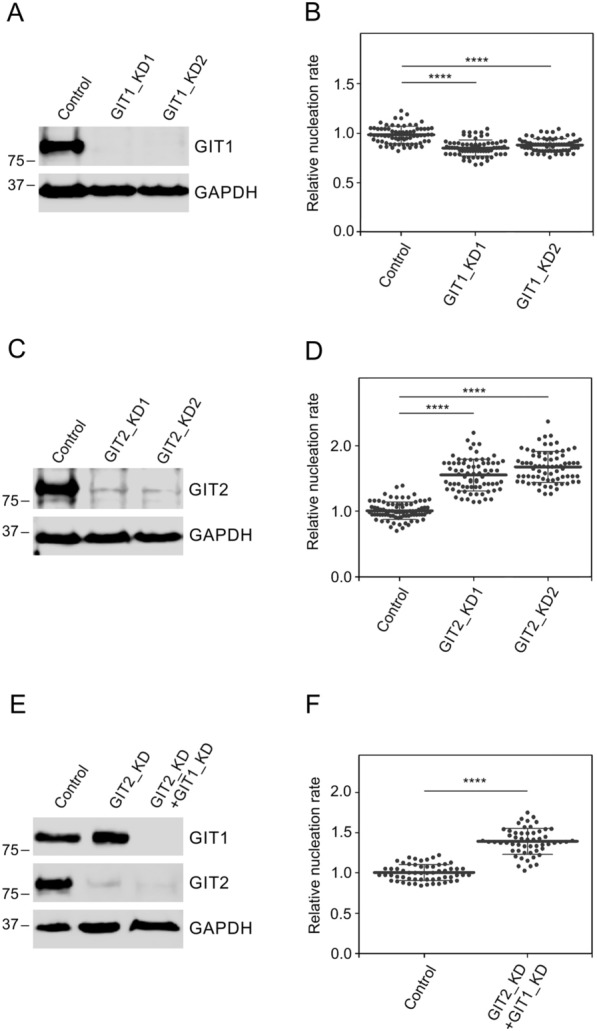
The effect of GIT1 and GIT2 depletion on centrosomal microtubule nucleation. Centrosomal microtubule nucleation in U-251 MG cells with reduced levels of GIT1 or GIT2, or both was analyzed using time-lapse imaging of EB3-NG. (**A**) Blots of whole-cell lysates probed with Abs to GIT1 and GAPDH (loading control). Control cells infected with the pLKO.1 vector containing non-target shRNA (Control), cells depleted of GIT1 by shRNA-GIT1-A (GIT1_KD1), or shRNA-GIT1-B (GIT1_KD2). (**B**) Comparison of the microtubule nucleation rate (EB3 comets/min) in GIT1_KD1 and GIT1_KD2 cells relative to control cells. Three independent experiments were conducted (at least 20 cells counted in each experiment): Control (pLKO.1-NT) (n = 72), GIT1_KD1 (n = 69), GIT1_KD2 (n = 70). (**C**) Blots of whole-cell lysates probed with Abs to GIT2 and GAPDH (loading control). Control cells infected with the pLKO.1 vector containing non-target shRNA (Control), cells depleted of GIT2 by shRNA-GIT2-A (GIT2_KD1), or shRNA-GIT2-B (GIT2_KD2). (**D**) Comparison of the microtubule nucleation rate (EB3 comets/min) in GIT2_KD1 and GIT2_KD2 cells relative to control cells. Three independent experiments were conducted (at least 19 cells counted in each experiment): Control (pLKO.1-NT) (n = 83), GIT2_KD1 (n = 75), GIT2_KD2 (n = 73). (**E**) Blots of whole-cell lysates probed with Abs to GIT1, GIT2, and GAPDH (loading control). Control cells infected with the pLKO.1 vector containing non-target shRNA (Control), cells depleted of GIT2 (GIT2_KD), and cells depleted of both GIT2 and GIT1 (GIT2_KD + GIT1_KD). **F** Comparison of the microtubule nucleation rate (EB3 comets/min) in cells with reduced levels of both GIT2 and GIT1 relative to control cells. Three independent experiments were conducted (at least 17 cells counted in each experiment): Control (pLKO.1-NT) (n = 58), GIT2_KD + GIT1_KD (n = 55). (**B**, **D**, **F**) The bold and thin lines within the dot plots represent mean ± SD. **B**, **D** A one-way ANOVA with Dunnett’s multiple comparison test was performed to determine statistical significance. (**F**) A two-tailed, unpaired Student’s *t*-test was performed to determine statistical significance. ****, p < 0.0001

To explore the involvement of GIT1 and GIT2 in centrosomal microtubule nucleation, we generated cell lines deficient in these proteins using lentiviral vectors. As a control, we employed cells with lentiviral vectors for non-targeting shRNA (designated as Control, PLKO.1-NT). A representative immunoblot illustrating GIT1 depletion is shown in Fig. 4A. Unless indicated otherwise, subsequent analyses are based on GIT1_KD1 cells, hereafter referred to as GIT1_KD. Live-cell imaging using EB3-mNeonGreen (EB3-NG) to track microtubule growth and counting the number of EB3 comets leaving centrosomes per unit time (nucleation rate) revealed a modest reduction in centrosomal microtubule nucleation in GIT1_KD1 and GIT1_KD2 cells relative to control cells (Fig. 4B). Similarly, a representative immunoblot demonstrating GIT2 depletion is presented in Fig. 4C, and unless stated otherwise, the following results pertain to GIT2_KD2 cells, hereafter referred to as GIT2_KD. In contrast to the effects observed in GIT1_KD cells, time-lapse imaging of GIT2_KD cells revealed a pronounced elevation in centrosomal nucleation in both GIT2_KD1 and GIT2_KD2 cells compared to control cells (Fig. 4D). This increase was apparent in both single-frame images and 10-frame projections of control and GIT2_KD2 cells (Fig. S5A). Simultaneous depletion of both GIT1 and GIT2 (Fig. 4E) resulted in increased microtubule nucleation (Fig. 4F). GIT1 and GIT2 therefore differentially regulate microtubule nucleation, with GIT2 playing a more prominent role in this process.

**Fig. 5:**
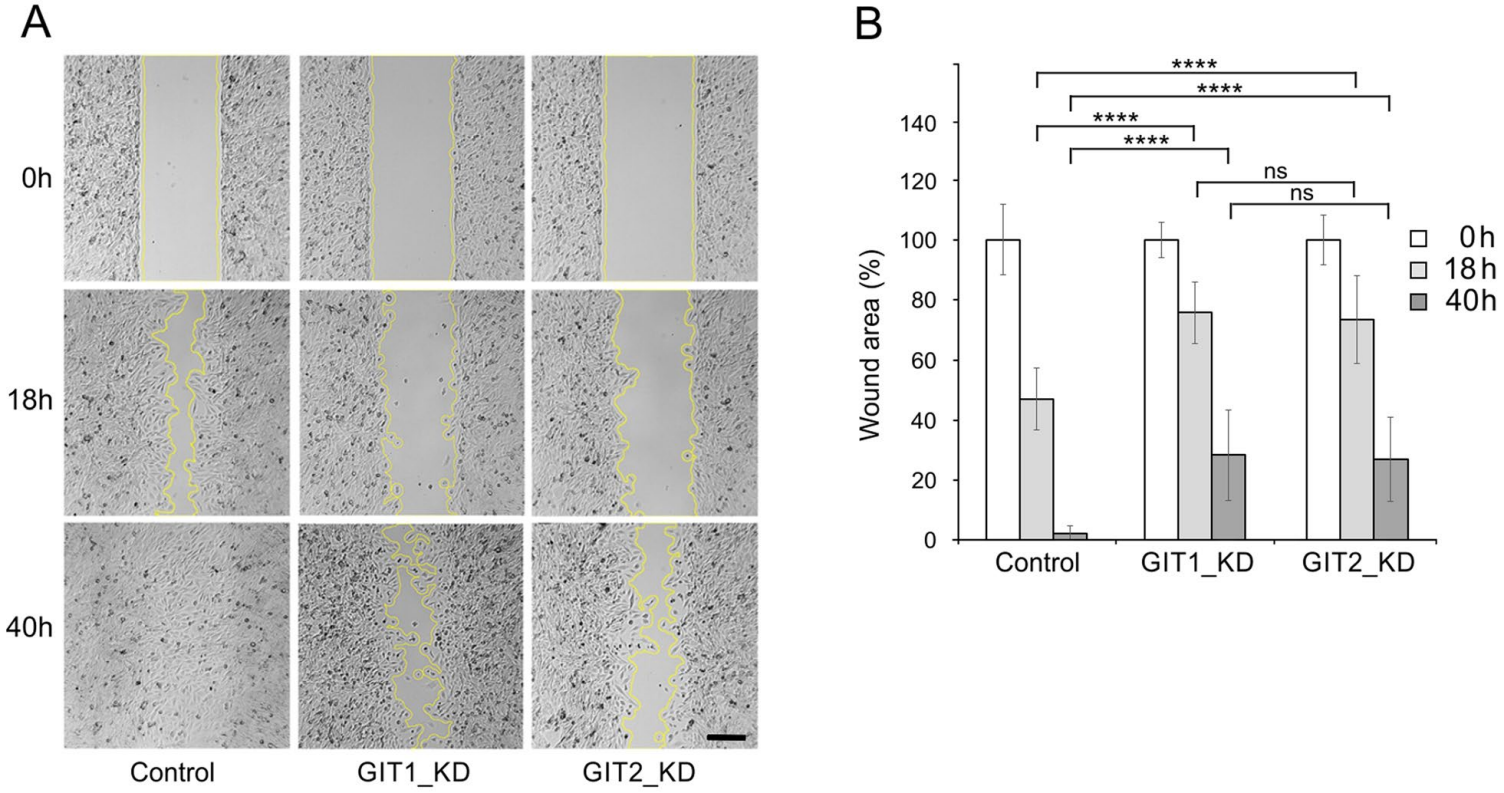
The effect of GIT1 and GIT2 depletion on cell migration. (**A**) Microscopy images showing wound closure in U-251 MG cells infected with the pLKO.1 vector containing non-target shRNA (Control, pLKO.1-NT) and cells with depleted levels of GIT1_KD or GIT2_KD at 0, 18, and 40 h. The yellow lines delineate the area devoid of cells. Scale bar, 250 μm. (**B**) Quantification of the wounded area invaded after 18 and 40 h in Controls, GIT1_KD, and GIT2_KD cells. Values represent mean ± SD (Control, n = 14; GIT1_KD, n = 16; GIT2_KD, n = 15). A two-tailed, unpaired Student’s *t*-test was performed to determine statistical significance. ns, p > 0.05; ****, p < 0.0001

To evaluate whether GIT1 and GIT2 also differentially regulate cell migration in U251 MG cells, we followed cell migration in control cells containing non-targeting shRNA (Control), and in GIT1_KD and GIT2_KD cells. For this, we used a wound healing assay. GIT1_KD and GIT2_KD showed different behavior when compared to control cells. While in control cells wound was substantially covered after 18 h and fully covered in 40 h, the migration of GIT1_KD and GIT2_KD was suppressed and even after 40 h a free space remained in GIT1_KD and GIT2_KD cells (Fig. 5A). Quantification of wound area disclosed that while there was significant difference between Control and GIT1_KD or GIT2_KD cells, there was no significant difference between GIT1_KD and GIT2_KD cells (Fig. 5B). Simultaneous depletion of both GIT1 and GIT2 yielded results similar to those observed for GIT1_KD or GIT2_KD alone.

Taken together, these findings indicate that GIT2 depletion has a more pronounced and opposite effect on microtubule nucleation when compared to GIT1. On the other hand depletion of GIT1 and GIT2 similarly decreased cell migration.

### ArfGAP domain of GIT2 affects centrosomal microtubule nucleation

As the depletion of GIT2 had a more pronounced effect on microtubule nucleation than the depletion of GIT1, we concentrated on the role of GIT2 in this process. The increase in microtubule nucleation following GIT2 depletion was not confined to U-251 MG cells, as a similar effect was observed in U-87 MG (Fig. S5B), and U-118 MG glioblastoma cells (Fig. S5C).

**Fig. 6:**
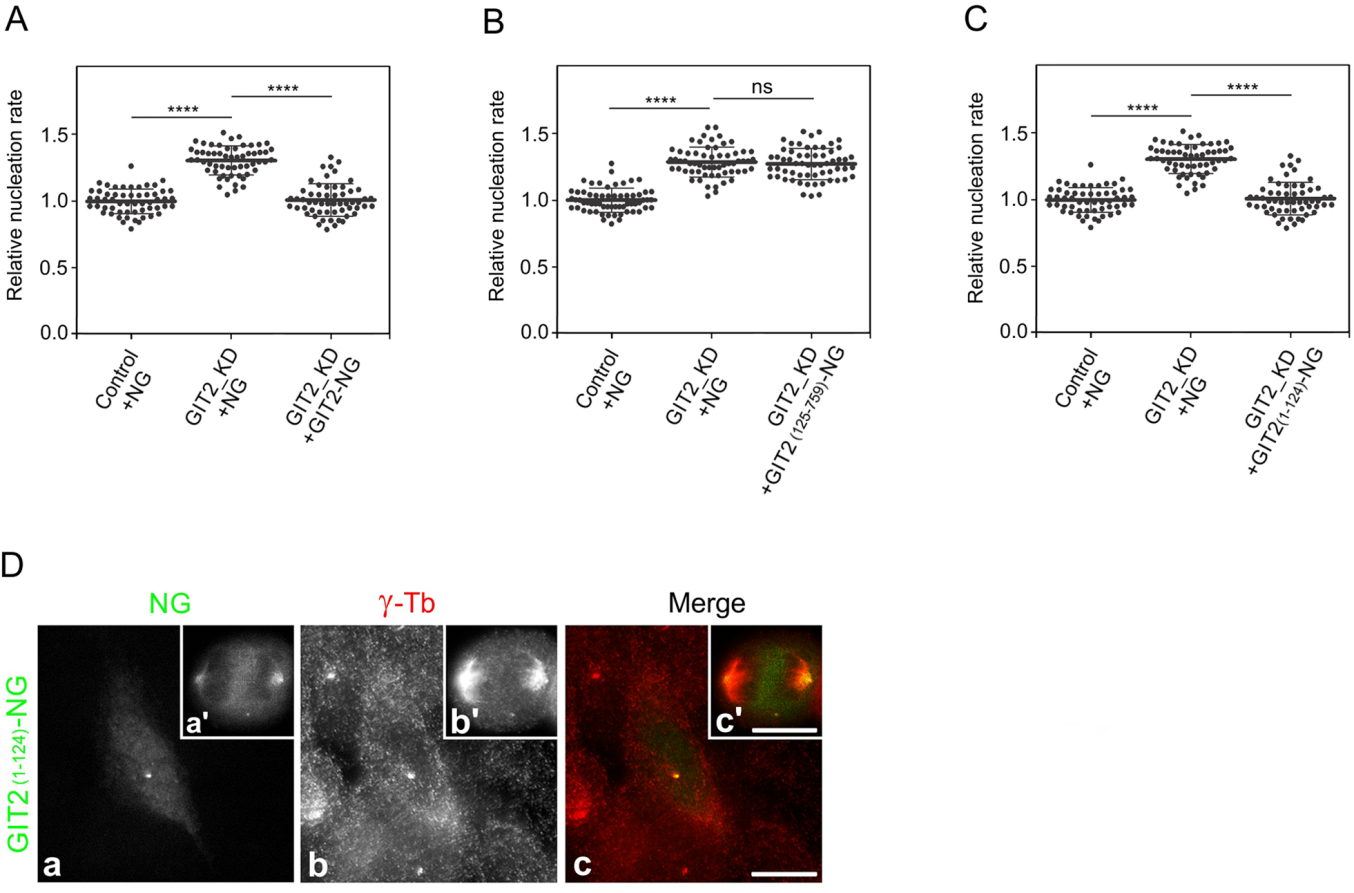
Phenotypic rescue of increased microtubule nucleation in GIT2-depleted cells and the association of the GIT2 N-terminal Arf GAP domain with centrosomes. (**A**) Comparison of the microtubule nucleation rate (EB3 comets/min) in GIT2_KD cells expressing NG and GIT2-NG, respectively, relative to control cells expressing NG. Three independent experiments were conducted (at least 15 cells counted in each experiment): Control + NG (n = 56), GIT2_KD + NG (n = 57), GIT2_KD + GIT2-NG (n = 57). (**B**) Comparison of the microtubule nucleation rate (EB3 comets/min) in GIT2_KD cells expressing NG and GIT2_(125–759)_-NG, respectively, relative to control cells expressing NG. Three independent experiments were conducted (at least 18 cells counted in each experiment): Control + NG (n = 62), GIT2_KD + NG (n = 61), GIT2_KD + GIT2_(125–759)_-NG (n = 60). (**C**) Comparison of the microtubule nucleation rate (EB3 comets/min) in GIT2_KD cells expressing NG and GIT2_(1–124)_-NG, respectively, relative to control cells expressing NG. Three independent experiments were conducted (at least 19 cells counted in each experiment): Control + NG (n = 62), GIT2_KD + NG (n = 60), GIT2_KD + GIT2_(1–124)_-NG (n = 62). (**A**–**C**) The bold and thin lines within the dot plots represent mean ± SD. A one-way ANOVA with Sidak’s multiple comparison test was performed to determine statistical significance. ns, p > 0.05; ****, p < 0.0001. (**D**) Association of the N-terminal Arf-GAP domain of GIT2 with interphase centrosomes and mitotic spindle poles in U-251 MG cells. Cells expressing GIT2_(1–124)_-NG were fixed and stained with Ab to γ-tubulin. GIT2_(1–124)_-NG (**a, a’**), γ-tubulin (**b, b’**; γ-Tb), and superposition of images (**c, c’**; GIT2_(1–124)_-NG in green and γ-tubulin in red). Cells in interphase are shown in panels **a-c**, and dividing cells in metaphase are shown in the insets **a’-c’**. Fixation Tx/F/M. Scale bars, 10 μm (**a-c, a’-c’**)

To validate the specificity of microtubule nucleation in GIT2_KD cells, we performed phenotypic rescue experiments in U-251 MG cells with depleted levels of GIT2, using the whole-length GIT2 or its truncated forms. First, we transfected GIT2-NG or NG alone into GIT2_KD cells (Fig. S5D). The introduction of GIT2-NG led to a decrease in microtubule nucleation, while the expression of NG alone did not have such an effect (Fig. 6A), confirming the specificity of increased nucleation after GIT2 depletion. When we introduced GIT2-NG without N-terminal ArfGAP domain (aa 1–124) into GIT2_KD cells (Fig. S5E; GIT2_(125–759)_-NG), we did not observe rescue of microtubule nucleation (Fig. 6B), suggesting the importance of N-terminal ArfGAP domain in the regulation of microtubule nucleation. Introduction of tagged ArfGAP domain into GIT2_KD cells (Fig. S5F; GIT2_(1–124)_-NG) directly confirmed that N-terminal domain of GIT2 participates in the regulation of microtubule nucleation (Fig. 6C). Fluorescence microscopy revealed that GIT2_(1–124)_-NG localized both to interphase centrosomes and spindle poles, but not to focal adhesions (Fig. 6D). This was expected as paxilllin binding region is located in the C-terminal part of GIT2 molecule [40].

To evaluate whether the substantial increase in microtubule nucleation in GIT2_KD cells is reflected in microtubule mass, we quantified polymerized microtubules by immunoblotting. For this, cells were extracted at 37°C with 0.2% Triton X-100 in a microtubule-stabilizing buffer containing 25% glycerol to ensure intact microtubules without depolymerizing into tubulin heterodimers. Microtubules with nuclei were then spun down at low speed, and the pelleted material was analyzed by immunoblotting with Abs to β-tubulin and nucleolin (major nucleolar protein; serving as a loading control). The β-tubulin signal, reflecting the microtubule mass, significantly increased in GIT2_KD cells compared to control cells (Fig. S5G).

Collectively, these findings suggest that the N-terminal ArfGAP domain of GIT2 plays an important role in regulating centrosomal microtubule nucleation. Additionally, the enhanced microtubule nucleation observed in GIT2-depleted cells leads to an increase in microtubule amount.

### PKC associates with GIT2 and regulates microtubule nucleation

**Fig. 7:**
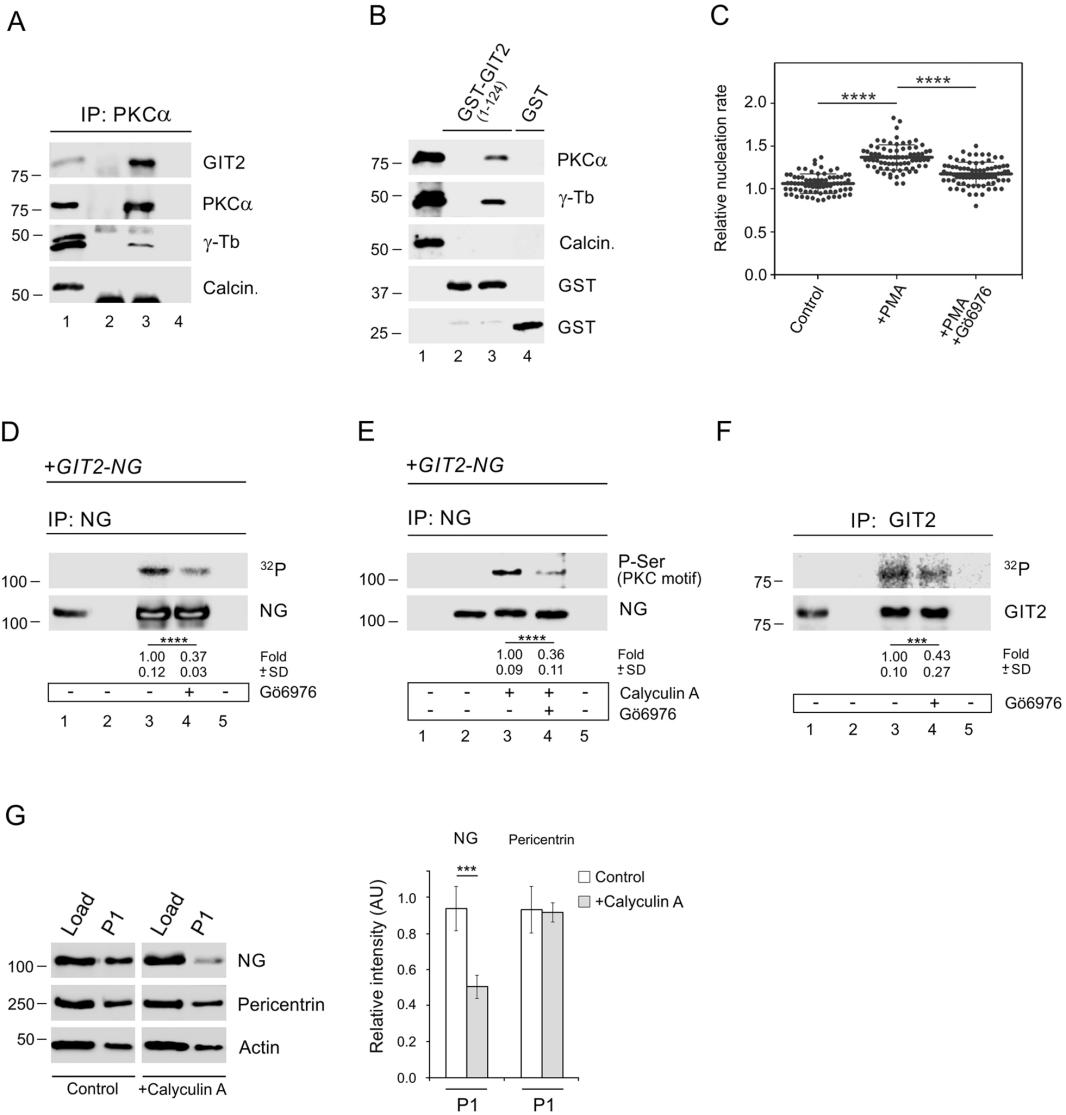
PKC modulates microtubule nucleation and phosphorylates GIT2. (**A**) Immunoprecipitation experiments using whole-cell extracts from U-251 MG cells and immobilized Ab to PKCα. The blots were probed with Abs to GIT2, PKCα, γ-tubulin (γ-Tb), and calcineurin (Calcin., negative control). Load (*lane 1*), immobilized Abs without cell extracts (*lane 2*), precipitated proteins (*lane 3*), and Ab-free carrier incubated with cell extract (*lane 4*). (**B**) GST-fusion protein GIT2_(1–124)_ or GST alone were immobilized on Glutathione-Sepharose and incubated with a whole-cell extract from U-251 MG cells. The blots were probed with Abs to PKCα, γ-tubulin (γ-Tb), calcineurin (Calcin., negative control), and GST. Load (*lane 1*), GST-fusion protein without cell extract (*lane 2*), proteins bound to GST-fusion protein (*lane 3*), and proteins bound to GST alone (*lane 4*). (**C**) Comparison of the microtubule nucleation rate (EB3 comets/min) in cells with activated PKCs, relative to control cells. Cells were pretreated with DMSO alone (Control), PKC activator PMA (1 μM for 30 min), or the PKC inhibitor Gö6976 (10 μM for 30 min) before adding PMA. Three independent experiments were conducted (at least 17 cells counted in each experiment): Control (n = 75), + PMA (n = 75), + PMA + Gö6976 (n = 77). The bold and thin lines within the dot plots represent mean ± SD. A one-way ANOVA with Sidak’s multiple comparison test was performed to determine statistical significance. ****, p < 0.0001. (**D**–**E**) Immunoprecipitation experiments with Abs to NG and whole-cell extracts from cells expressing GIT2-NG. (**D**) Kinase assay following immunoprecipitation (^32^P). Load (*lane 1*), immobilized Ab without cell extract (*lane 2*), precipitated proteins (*lanes 3–4*), and Ab-free carrier incubated with cell extract (*lane 5*). Cells were pretreated with DMSO alone (*lane 3*) or with the PKC inhibitor Gö6976 (10 μM for 30 min; *lane 4*). The blot was probed with Ab to NG. The numbers under the blot indicate the relative amounts of phosphorylated GIT2-NG (^32^P) normalized to Gö6976 untreated cells and the amount of precipitated GIT2-NG in individual samples (fold). Mean ± SD (n = 7). (**E**) Phosphorylation of GIT2-NG by PKC. Immobilized Ab without cell extract (*lane1*), precipitated proteins (*lanes 2–4*), and Ab-free carrier incubated with cell extract (*lane5*). Cells were pretreated with DMSO alone (*lane 3*) or with the PKC selective inhibitor Gö6976 (10 μM for 30 min; *lane 4*) before incubation with calyculin A (80 nM for 30 min). The blots were probed with Abs to P-Ser in the PKC motif and NG. The numbers under the blot indicate the relative amounts of GIT2-NG phosphorylated on serine (P-Ser in the PKC motif) normalized to Gö6976-untreated cells and the amount of precipitated GIT2-NG in individual samples (fold). Mean ± SD (n = 5). (**F**) Kinase assay after immunoprecipitation experiment with Ab to GIT2 and whole-cell extract (^32^P). Load (*lane 1*), immobilized Ab without cell extract (*lane 2*), precipitated proteins (*lanes 3–4*), and Ab-free carrier incubated with cell extract (*lane 5*). Cells were pretreated with DMSO alone (*lane 3*) or with the PKC inhibitor Gö6976 (10 μM for 30 min; *lane 4*). The blot was probed with Ab to GIT2. The numbers under the blot indicate the relative amounts of phosphorylated GIT2 (^32^P) normalized to Gö6976-untreated cells and the amount of precipitated GIT2 in individual samples (fold). Mean ± SD (n = 6). (**G**) Relative distribution of proteins in fractions after differential centrifugation of homogenates from GIT2_KD + GIT2-NG cells. Cell fractions were prepared as described in the Materials and Methods section. Cell homogenate (*lane 1*), pellet P1 (*lane 2*). Immunoblot analysis was performed with Abs to NG, pericentrin, and actin (loading control). Densitometric quantification of immunoblots is shown on the right. Intensities of corresponding proteins in P1 normalized to loads (relative intensity 1.0). Mean ± SD (n = 4). (**D-G**) A two-tailed, unpaired Student’s *t*-test was performed to determine statistical significance. ***, p < 0.001; ****, p < 0.0001

As depletion of GIT2 had a pronounced effect on microtubule nucleation, we asked whether phosphorylation of GIT2 can be involved in the regulation of this process. Previously we have shown that Ca^2+^ and diacylglycerol-regulated conventional PKCs (cPKCs) modulate centrosomal microtubule nucleation [22] in mast cells. There are four isoforms of cPKCs, namely PKCα, PKCβI, PKCβII, and PKCγ [41]. To investigate whether cPKCs affect microtubule nucleation in glioblastoma cells, we first evaluated the expression profile of cPKCs in U-251 MG cells using gel-based RT-PCR analysis. We found that the PKCα isoform was the most prominent, while the other cPKC isoforms were under the detection limit (Fig. S5H). Immunoprecipitation experiments revealed immunocomplexes containing PKCα, GIT2, and γ-tubulin (Fig. 7A). Pull-down experiments then disclosed the association of both PKCα and γ-tubulin with GST-tagged N-terminal ArfGAP domain of GIT2 (Fig. 7B; GST-GIT2_(1–124)_). To activate PKCs in U-251 MG cells, we pretreated cells with PMA, which stimulates conventional and novel PKCs by mimicking the action of diacylglycerol [42]. Activation of PKCs resulted in a significant increase of microtubule nucleation and this effect was suppressed in the presence of PKC inhibitor Gö6976 that preferentially targets cPKCs [43] (Fig. 7C). To assess whether exogenous GIT2 can be phosphorylated by PKC, we pretreated cells expressing GIT2-NG with Gö6976 or DMSO carrier alone and performed an in vitro kinase assay after immunoprecipitation using whole-cell extracts and the anti-NG Ab. In vitro kinase assay revealed that phosphorylation of exogenous GIT2-NG was inhibited in the presence of Gö6976 inhibitor (Fig. 7D). Similar inhibitory effect was observed when the other PKC specific inhibitors sotrastaurin and Gö6983 were applied. To independently confirm phosphorylation of GIT2-NG by PKCs, we treated cells expressing GIT2-NG with calyculin A, a potent inhibitor of Ser/Thr phosphatases. After immunoprecipitation with the anti-NG Ab, the blots were probed with Ab recognizing P-Ser in PKC motifs. Exogenous GIT2 was phosphorylated by PKCs and phosphorylation was reduced by Gö6976. (Fig. 7E). Finally, the in vitro kinase assay after immunoprecipitation with anti-GIT2 Ab confirmed that also endogenous GIT2 was phosphorylated and its phosphorylation was inhibited by PKC inhibitor (Fig. 7F). To assess how phosphorylation affects the subcellular localization of GIT2, we used GIT2_KD cells expressing GIT2-NG. Pretreating these cells with calyculin A reduced the level of GIT2-NG in the P1 fraction, which contains centrosomes and nuclei, compared to the untreated control cells. However, the amount of pericentrin in the P1 fractions remained similar between the control and calyculin A-treated cells (Fig. 7G).

These results indicate that both endogenous and exogenous GIT2 are phosphorylated by PKCs, which may impact GIT2 conformation or its subcellular localization. Furthermore, PKCs play a role in regulating centrosomal microtubule nucleation.

### Identification of phosphorylation site for PKCα in the ArfGAP domain of GIT2

**Fig. 8:**
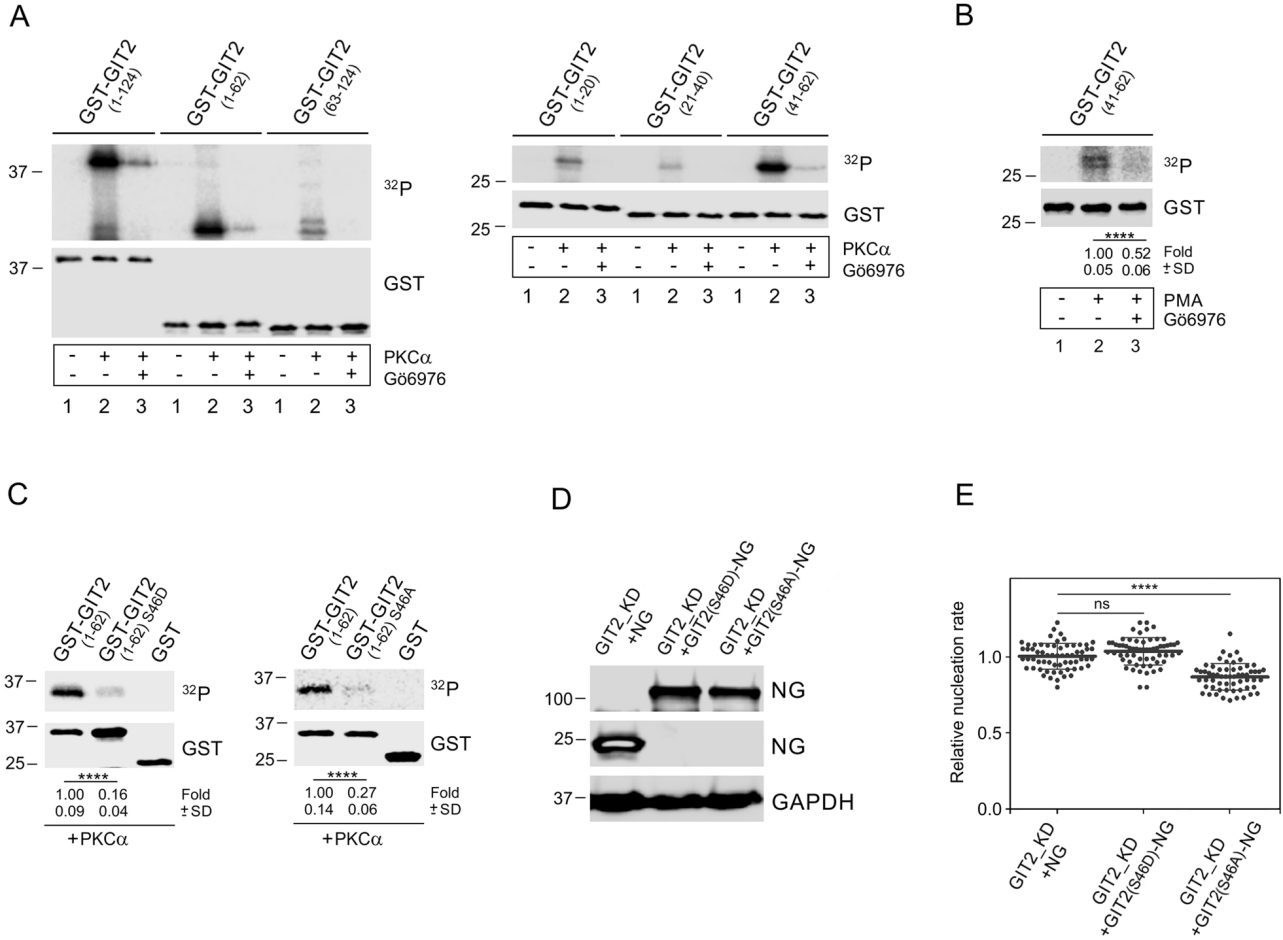
PKCα phosphorylates S46 in the Arf-GAP domain of GIT2. Phosphorylation of S46 affects microtubule nucleation. (**A**) Phosphorylation of the Arf-GAP domain of GIT2 and its truncated variants by PKCα. Immobilized GST-tagged Arf-GAP domain GST-GIT2_(1–124)_ and its truncated versions GIT2_(1–62)_, GIT2_(63–124)_, GIT2_(1–20)_, GIT2_(21–40)_, GIT2_(41–62)_ were subjected to a kinase assay with active PKCα in the absence or presence of the PKC inhibitor Gö6976 (10μM). Phosphorylated proteins were detected by autoradiography (^32^P), and the blots were probed with Ab to GST. GST-fusion proteins without PKCα (*lane 1*), with PKCα (*lane 2*), and with PKCα and Gö6976 (*lane 3*). (**B**) Kinase assay after pull-down with immobilized GST-GIT2_(41–62)_ and whole-cell extract. Phosphorylated proteins were detected by autoradiography (^32^P), and the blot was probed with Ab to GST. GST-fusion protein alone (*lane 1*) and with cell extracts (*lanes 2–3*). Extract from cell treated with PMA (*lane 2*), extract from cells treated with PMA and Gö6976 (*lane 3*). The numbers under the blot indicate the relative amounts of phosphorylated GIT2-NG_(41–62)_ (^32^P), normalized to Gö6976 untreated cells and the amount of GST-GIT2-NG_(41–62)_ in individual samples (fold). Mean ± SD (n = 5). (**C**) Phosphorylation of phosphomimetic (S46D) and non-phosphorylatable (S46A) mutations of GST-GIT2_(1–62)_. GST-GIT2_(1–62)_, along with its phosphomimetic or non-phosphorylatable variants at the position of S46, and GST alone were immobilized and subjected to a kinase assay with active PKCα. Phosphorylated proteins were detected by autoradiography, and the blot was probed with Ab to GST. The numbers under the blot indicate the relative amounts of phosphorylated GST-tagged mutants (^32^P), normalized to phosphorylated GST-GIT2_(1–62)_ and the amount of GST-tagged proteins in each sample (fold). Mean ± SD (n = 8 for both S46D and S46A). (**B**–**C**) A two-tailed, unpaired Student’s *t*-test was performed to determine statistical significance. ****, p < 0.0001. (**D**) Immunoblot analysis of whole-cell lysates from GIT2_KD cells expressing NG (GIT2_KD + NG), and GIT2_KD cells rescued by GIT2_(S46D)_-NG or GIT2_(S46A)_-NG. The blots were probed with Abs to NG and GAPDH (loading control). (**E**) Comparison of microtubule nucleation rate (EB3 comets/min) in GIT2_KD cells expressing GIT2_(S46D)_-NG and GIT2_(S46A)_-NG, relative to GIT2_KD cells expressing NG. Three independent experiments were conducted (at least 20 cells counted in each experiment): GIT2_KD + NG (n = 63), GIT2_KD + GIT2_(S46D)_-NG (n = 61), and GIT2_KD + GIT2_(S46A)_-NG (n = 60). The bold and thin lines within the dot plots represent mean ± SD. A one-way ANOVA with Dunnett’s multiple comparison test was performed to determine statistical significance. ns, p > 0.05; ****, p < 0.0001

Because the ArfGAP domain of GIT2 (GIT2_1–124_) plays a modulatory role in centrosomal microtubule nucleation (Fig. 6C), and PKCs modulate microtubule nucleation (Fig. 7C), we asked whether the ArfGAP domain could by phosphorylated by PKCα. The kinase assay using GST-tagged GIT2(_1–124_) and purified active recombinant PKCα revealed that PKCα phosphorylates the ArfGAP domain (Fig. 8A). In contrast, the active control PAK1, which phosphorylates GIT1 at S709 [44] and S519 [20], did not target the ArfGAP domain of GIT2 (Fig. S5I). The kinase assay using truncated versions of ArfGAP domain—specifically GST-tagged GIT2_(1–62)_, GIT2_(63–124)_, GIT2_(1–20)_, GIT2_(21–40)_, and GIT2_(41–62)_—and active PKCα revealed that PKCα dominantly targeted the 41–62 aa region of the ArfGAP domain (Fig. 8A). The pull-down experiments with GST-GIT2_(41–62)_ and cell lysates followed by in vitro kinase assays, revealed that in the presence PMA, the phosphorylation of GST-tagged protein was augmented and that increased phosphorylation was attenuated by Gö6976 inhibitor. This suggests that the ArfGAP domain in the 41–62 aa region is phosphorylated by cellular PKCs (Fig. 8B). Using the PhosphoNet Kinase Predictor Tool from Kinexus (Vancouver, BC, Canada), we identified the S46 position in the 41–62 aa region as a potential phosphorylation site for PKCs. Kinase assays with phosphomimetic (S46D) and non-phosphorylatable (S46A) mutations of GST-GIT2_(1–62)_ and active PKCα showed substantially reduced phosphorylation by PKCα suggesting that S46 can be targeted by PKCα (Fig. 8C). To assess the impact of S46 phosphorylation on microtubule nucleation, we mutated S46 in whole-length GIT2 tagged with NG. Introduction of GIT2_(S46D)_-NG, GIT2_(S46A)_-NG, or NG alone into cells with depleted levels of GIT2 (Fig. 8D) revealed, that phosphomimetic (S46D) mutation or NG alone were not capable of rescuing microtubule nucleation, while non-phosphorylatable mutation (S46A) suppressed microtubule nucleation (Fig. 8E). This indicates that phosphorylation of GIT2 on S46 plays a regulatory role in centrosomal microtubule nucleation.

Altogether, these data suggest that phosphorylation of S46 on GIT2, which can be targeted by PKCα, promotes centrosomal microtubule nucleation.

## Discussion

Previously, we demonstrated that GITs regulate microtubule nucleation in mast cells. This led us to investigate whether GITs might similarly regulate microtubule nucleation in glioblastomas, which are marked by dysregulated expression of γ-tubulin [45], a key component of γTuRCs essential for the nucleation process. In glioblastoma cell lines overexpressing γ-tubulin, prominent staining for γ-tubulin has been observed not only in centrosomes but also in the cytoplasm [23, 45]. Unlike αβ-tubulin dimers, which are encoded by multiple gene isotypes and undergo extensive post-translational modification in brain tissue [46, 47], γ-tubulin has only two isotypes that are modified less extensively [8, 25]. In this study, we demonstrate that both γ-tubulin isotypes are significantly overexpressed in three human glioblastoma cell lines compared to normal human astrocytes. GIT1 and GIT2 both associate with γTuRC proteins and localize to centrosomes, but they play opposing roles in microtubule nucleation, with GIT2 having a more pronounced effect. The N-terminal ArfGAP domain of GIT2 associates with centrosomes, regulates microtubule nucleation, and can be phosphorylated by PKC. We show that phosphorylation of the S46 site in the ArfGAP domain affects microtubule nucleation. These findings suggest a novel regulatory mechanism for microtubule nucleation in glioblastoma cells.

Several lines of evidence highlight the specific role of GIT1 and GIT2 in regulating centrosomal microtubule nucleation. First, reciprocal immunoprecipitation experiments demonstrated that both endogenous and exogenous GIT1 and GIT2 associate with γTuRC proteins. Second, pull-down assays using whole-cell extracts and GST-tagged GIT1, GIT2, and γ-tubulin confirmed that γTuRC proteins interact with GITs and that GITs associate with γ-tubulin. Third, isolated functional centrosomes were found to contain GIT1 and GIT2. Fourth, NG-tagged versions of both GIT1 and GIT2 localized to centrosomes. Finally, depletion of GIT1 moderately inhibited microtubule nucleation, while GIT2 depletion strongly enhanced it. These findings suggest that GIT1 and GIT2 play different roles in the regulation of microtubule nucleation in glioblastoma cells. The impact of GIT1 depletion on microtubule nucleation was significantly less pronounced in glioblastoma cells compared to mouse mast cells [21] or human osteosarcoma cells [28]. In contrast, GIT2 depletion had a similarly strong effect in both mast cells [22] and in glioblastoma cells, leading to an increase in microtubule amount. In mast cells, we demonstrated that GIT1 could not rescue the increased microtubule nucleation in cells lacking GIT2 [22]. This suggests that while GIT1 and GIT2 play distinct roles in centrosomal microtubule nucleation, their effects may vary depending on the cell type, with GIT2 exhibiting a more consistent role across different cell types in various species. Regulating the balance of GITs activities at the centrosome may modulate microtubule nucleation, potentially affecting the number of microtubules available for intracellular transport and other cellular activities. It is important to note that other GAPs for Arf family GTPases [15] could also play a role in microtubule nucleation. For example, ELMOD2, a GAP for the Arf-like GTPase Arl2, interacts with γTuRCs, and its deletion disrupts the recruitment of γTuRCs to centrosomes, impairing microtubule nucleation [48, 49]. Additionally, Arl2 has been shown to regulate centrosomal microtubule dynamics in various cell types [50] and can physically associate with CDK5RAP2 [51], a key protein for centrosomal targeting and activation of γTuRCs (Sulimenko et al. 2022).

The different regulatory roles of GIT1 and GIT2 in microtubule nucleation are highlighted by differential subcellular distribution of their tagged variants in diverse glioblastoma cell lines. GIT1 localizes primarily to focal adhesions and centrosomes, whereas GIT2 predominantly localizes to centrosomes, with a weaker association with focal adhesions. It was reported that GIT2 binds to paxillin with much lower affinity than GIT1 [16] and that GIT2, but not GIT1, is an essential inhibitor of cell spreading and focal adhesion turnover regulated by Rac1 and Cdc42, respectively [52]. Another difference is that GIT1 and GIT2 regulate distinct signaling pathways during chemotaxis in RBL-2H3 cells [53]. Furthermore, GIT1 and GIT2 have been shown to influence synaptic strength in neurons by enhancing exocytosis efficiency via separate mechanisms [54]. These findings suggest that, despite their structural similarities, GIT1 and GIT2 may perform distinct cellular functions, including differential regulation of microtubule nucleation.

While the depletion of GIT1 and GIT2 has diverse effects on centrosomal microtubule nucleation, the depletion of either GIT1 or GIT2 resulted in the inhibition of glioblastoma cell migration, as demonstrated by a wound healing assay. GIT1 and GIT2 are key regulators of focal adhesion turnover and cell motility in cancer cells [17]. The depletion of GIT1 in highly aggressive breast cancer, oral squamous cancer, and lung cancer cell lines results in a significant loss of invasive properties, as reviewed by Yoo et al. [55]. Similarly, GIT2 depletion in lung cancer cell lines inhibited cell migration [56]. In contrast, the depletion of GIT2 in non-transformed epithelial cells was reported to stimulate cell migration [52, 57]. In our previous studies on mast cells, the depletion of GIT1 enhanced chemotaxis [21], while the depletion of GIT2 inhibited chemotaxis to antigen [22]. The differences noted in various studies could be due to variations in experimental cell models, as it is increasingly clear that the impact of GIT1 and GIT2 on cell migration depends on the specific cell type and on the signaling machinery responsible for sensing direction [58].

Since the depletion of GIT2 had a more significant impact on microtubule nucleation compared to GIT1, we focused on the role of GIT2 in this process. Phenotypic rescue experiments demonstrated that the impact of GIT2 depletion on microtubule nucleation could be restored by either full-length GIT2 or its N-terminal ArfGAP domain (aa 1–124). In contrast, the remaining portion of the GIT2 molecule (aa 125–759) was ineffective in rescuing nucleation. Additionally, the tagged GIT2_(1–124)_ localized to interphase centrosomes and spindle poles during mitosis. These results underscore the important role of the ArfGAP domain of GIT2 in the modulation of centrosomal microtubule nucleation. Previous studies have shown that the aa region 1–119 of the ArfGAP domain in GIT1 serves as a centrosome-targeting region [20]. Additionally, we demonstrated the localization of GFP-GIT1_(1–124)_ to the centrosomes of human osteosarcoma cells [28]. The Arf GAP domains of GIT proteins, which contain a zinc finger motif, bind not only Arf small GTPases to catalyze the hydrolysis of GTP on GDP [59], but also γ-tubulin as revealed by pull-down experiments using whole cell extracts and GST-GIT1_(1–124)_ [28] or GST-GIT2_(1–124_), as shown in this report. Together, these findings highlight the role of ArfGAP domains in guiding both GIT1 and GIT2 to centrosomes, and their ability to interact with γ-tubulin.

Our previous research demonstrated that cPKCs influence microtubule nucleation in mast cells [22]. In this study, we extend this observation to glioblastoma cells, where PKC stimulation by PMA promoted microtubule nucleation. Moreover, both exogenous and endogenous GIT2 were phosphorylated by PKCs. In our search for potential phosphorylation sites for cPKCs in the ArfGAP domain of GIT2, we used truncated forms of this domain and PKCα, the most abundant isoform of cPKCs at the transcript level in U-251 MG cells. We identified the S46 site, whose phosphorylation plays an important role in the modulation of microtubule nucleation, as confirmed by phosphomimetic (S46D) and non-phosphorylatable (S46A) mutations. The formation of PKCη–GIT2-αPIX-PAK complex was previously described in T_reg_ cells [60]. The S46 site is conserved in both GIT2 and GIT1, and it has previously been shown to be phosphorylated in GIT1 by the PKD3 kinase [61]. Since the sequences flanking S46 differ between GIT1 and GIT2, these sites may be targeted by distinct kinases in each protein. On the other hand, PKD3 itself is activated by phosphorylation through PKC isoforms [62]. Therefore, it remains unclear whether the role of cellular PKCs in the phosphorylation of the S46 site in the ArfGAP domain GIT2 is direct or indirect. The important roles of PKC isoforms in centrosome functions have been previously reported. Among the conventional PKCs, PKCβII has been found to interact with the centrosomal protein pericentrin in various cells, and the disruption of this interaction resulted in the disorganization of centrosomal microtubules. However, the target of pericentrin-tethered PKCβ remains unidentified [63]. Additionally, accumulation of PKCβI at the centrosomes has been observed in T lymphocytes treated with PMA [64], and in T lymphocytes activated by CD44 receptor crosslinking, PKCβ relocates to the centrosomes [65]. Furthermore, among the novel PKCs, PKCε regulates mitotic progression by controlling centrosome migration in pre-mitotic transformed cells [66], and PKCθ localizes to centrosomes during mitosis in multiple cell types, suggesting a role in cell proliferation [67]. Microtubules play a key role in the migration of cancer cells exhibiting a mesenchymal migration phenotype [68], which is characteristic of mesenchymal glioblastoma [69]. In glioblastomas, which overexpress PKCα compared to low-grade astrocytomas [70, 71], PKC-mediated phosphorylation of centrosomal GIT2 may enhance microtubule nucleation, thereby promoting the invasive properties of malignant cells.

Phosphorylation of GIT2 by PKC might affect its association with centrosomes and thus modulate microtubule nucleation. We have shown that pretreatment of cells with calyculin A, an inhibitor of Ser/Thr phosphatases, resulted both in phosphorylation of tagged GIT2 and its partial dissociation from centrosomal fraction. It remains, however, unclear whether the subcellular relocation of GIT2 is due to PKC-mediated phosphorylation of GIT2 or other centrosomal protein(s). Alternatively, phosphorylation of the S46 site on centrosomal GIT2 could induce a conformation change in the protein, which might modulate its interaction with specific protein partners thereby affecting microtubule nucleation. GIT proteins undergo differential phosphorylation by various kinases [19]. P21-activated kinase 1 (PAK1) has been shown to phosphorylate GIT1 at S517, a site located in a region where GIT1 and GIT2 exhibit notable sequence divergence [20]. Tyrosine phosphorylation is essential for inducing intramolecular conformational changes in GIT1, leading to the release of its autoinhibitory interaction [72]. Phosphorylation of GIT2 at tyrosine residues Y286, Y392, and Y592 by Src/FAK is crucial for its binding to paxillin at focal adhesions [73]. Additionally, phosphorylation at Y392 promotes the interaction of GIT2 with the SH2-SH3 adaptor proteins Nck1 and Nck2 [74]. Given the key role of phosphorylation in regulating GIT protein functions [17], the distinct regulation of centrosomal microtubule nucleation by GIT1 and GIT2 may, in part, result from the differential phosphorylation of these GIT isoforms.

## Conclusions

Altogether, our results suggest a novel regulatory mechanism of microtubule organization in glioblastoma cells, where the phosphorylation of the S46 in the ArfGAP domain of GIT2 by PKCs promotes microtubule nucleation, supporting the invasive properties of these highly malignant cells.

## Supplementary Information

Supplementary material 1: This manuscript has supplementary material

## Data Availability

No datasets were generated or analysed during the current study.

## Abbreviations

Ab(s): Antibody(ies)
cPKCs: Ca^2+^- and diacylglycerol-regulated conventional protein kinases C
GCP: γ-Tubulin complex protein
GIT: G protein-coupled receptor kinase-interacting protein
GITs: GIT proteins
mAb: Mouse monoclonal antibody
NG: mNeonGreen
PAK: p21 protein (Cdc42/Rac)-activated kinase 1
PIX: P21-activated kinase interacting exchange factor
PMA: Phorbol 12-myristate 13-acetate
γTuRCs: γ-Tubulin ring complex
