# Supplementary material for: Regulation of microtubule nucleation in glioblastoma cells by ARF GTPase-activating proteins GIT1 and GIT2 and protein kinase C
Source: Cancer Cell Int. 2025 Apr 2;25:125. doi: 10.1186/s12935-025-03740-y (PMC11963297; doi:10.1186/s12935-025-03740-y)
Supplement: Supplementary file 1 — Supplementary material 1: This manuscript has supplementary material [file 12935_2025_3740_MOESM1_ESM.docx]

**Supplementary material**

**Table S1 Sequences of oligonucleotides used for the preparation of shRNA constructs for the depletion of human GIT1 or GIT2, and for the negative control (pLKO.1-NT)**

shRNA-GIT1-A

| Oligonucleotide | Sequence |
| --- | --- |
| Sense | 5’- CCGGAGCCTTGACTTATCCGAATTGCTCGAGCAATTCGGATAAGTCAA  GGCTTTTTTG -3’ |
| Antisense | 5’- AATTCAAAAAAGCCTTGACTTATCCGAATTGCTCGAGCAATTCGGATA  AGTCAAGGCT -3’ |

shRNA-GIT1-B

| Oligonucleotide | Sequence |
| --- | --- |
| Sense | 5’- CCGGGCTCTCCCTTTAATGCCATATCTCGAGATATGGCATTAAAGGGA  GAGCTTTTTG -3’ |
| Antisense | 5’- AATTCAAAAAGCTCTCCCTTTAATGCCATATCTCGAGATATGGCATTA  AAGGGAGAGC -3’ |

shRNA-GIT2-A

| Oligonucleotide | Sequence |
| --- | --- |
| Sense | 5’- CCGGATAACGGTGCTAACTCTATATCTCGAGATATAGAGTTAGCACCG  TTATTTTTTG -3’ |
| Antisense | 5’- AATTCAAAAAATAACGGTGCTAACTCTATATCTCGAGATATAGAGTTA  GCACCGTTAT -3’ |

shRNA-GIT2-B

| Oligonucleotide | Sequence |
| --- | --- |
| Sense | 5’- CCGGGAGTACTCATCAACACGAAATCTCGAGATTTCGTGTTGATGAGT  ACTCTTTTTG -3’ |
| Antisense | 5’- AATTCAAAAAGAGTACTCATCAACACGAAATCTCGAGATTTCGTGTTG  ATGAGTACTC -3’ |

shRNA-pLKO.1-NT

| Oligonucleotide | Sequence |
| --- | --- |
| Sense | 5’- CCGGCAACAAGATGAAGAGCACCAACTCGAGTTGGTGCTCTTCATCTT  GTTGTTTTTG -3’ |
| Antisense | 5’- AATTCAAAAACAACAAGATGAAGAGCACCAACTCGAGTTGGTGCTCTT  CATCTTGTTG -3’ |

Targeted sequences are underlined.

**Table S2 Sequences of primers used for the analysis of human genes**

Name Sequence Amplicon length

___________________________________________________________________________

*ACTB*, fwd 5‘-ATGTGGCCGAGGACTTTGATT-3‘ 106 bp

*ACTB*, rev 5‘-AGTGGGGTGGCTTTTAGGATG-3‘

*PRKCA,* fwd 5‘-CCAAAGTGTGTGGCAAAG-3‘ 109 bp

*PRKCA,* rev 5‘-TCAGACTGGTCTATGTTAGC-3‘

*PRKCB*, fwd 5’-GAGATAATTGCTTATCAGCCC-3’ 118 bp

*PRKCB*, rev 5’-AGAGTTCATCTTCATCCTCC-3’

*PRKCG*, fwd 5’-AATCAGGGCATCATCTACAG-3’ 78 bp

*PRKCG,* rev 5’-GTCAGTGATCTTGATGTGTC-3’

*TUBG1*, fwd 5‘-CCCTCATCTGCCTTACTGGTTG-3‘ 72 bp

*TUBG1*, rev 5‘-AGGTCCCTGATCTGTGCTCTGA-3‘

*TUBG2*, fwd 5’-GGAGCTCATTGATGAGTACCATG-3’ 95 bp

*TUBG2*, rev 5’-AGGAGAAGGAGTAGTGGGGAG-3’

___________________________________________________________________________


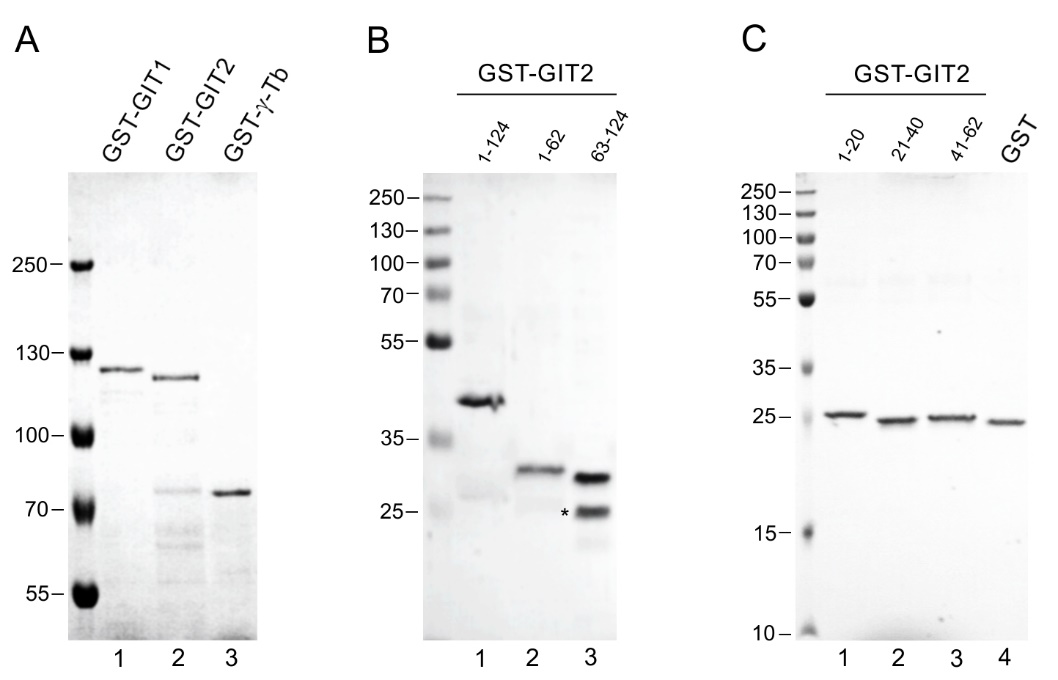


**Fig. S1** Coomassie Blue staining of GST-tagged proteins. (**A**) GST-GIT1 (lane 1), GST-GIT2 (lane 2), and GST-γ-tubulin (lane 3) separated on 7.5% SDS-PAGE. **(B**) GST-tagged Arf-GAP domain GST-GIT2_(1-124)_ (lane 1) and its truncated versions GST-GIT2_(1-62)_ (lane 2), and GST-GIT2_(63-124)_ (lane3) separated on 10% SDS-PAGE. (**C**) GST-GIT2_(1-20)_ (lane 1), GST-GIT2_(21-40_) (lane 2), GST-GIT2_(42-62)_ (lane 3), and GST alone (lane 4) separated on 12.5% SDS-PAGE. Asterisk (*) in panel B denotes proteolytic fragment of GST-GIT2_(63-124)_.


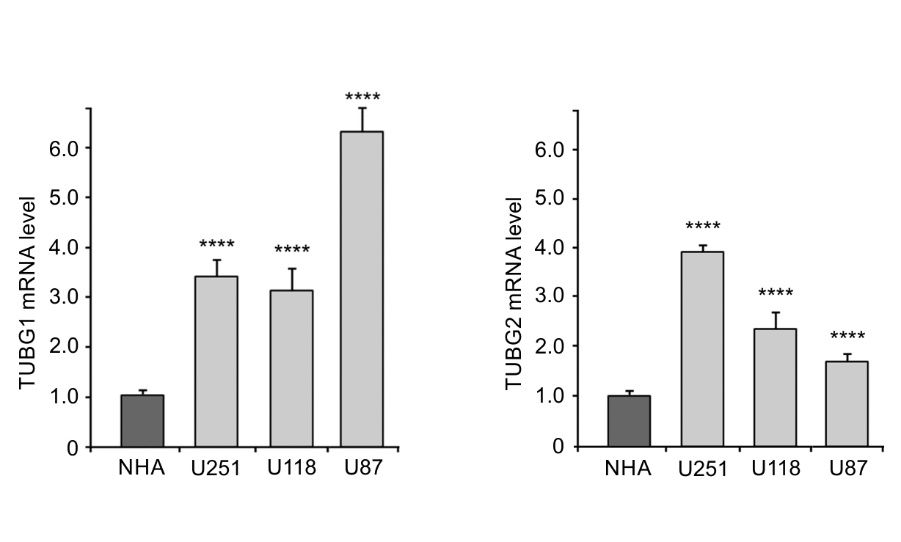


**Fig. S2** Comparison of γ-tubulin expression in normal human astrocytes and glioblastoma cell lines. Transcription levels of genes for γ-tubulin 1 (*TUBG1*) and γ-tubulin 2 (*TUBG2*) in glioblastoma cell lines U-251 MG (U251), U-118 MG (U118), and U-87 MG (U87) were measured relative to those in normal human astrocytes (NHA). Data are presented as mean fold change ± SD (n=4). A two-tailed, unpaired Student’s *t*-test was performed to determine statistical significance. ****, p < 0.0001.


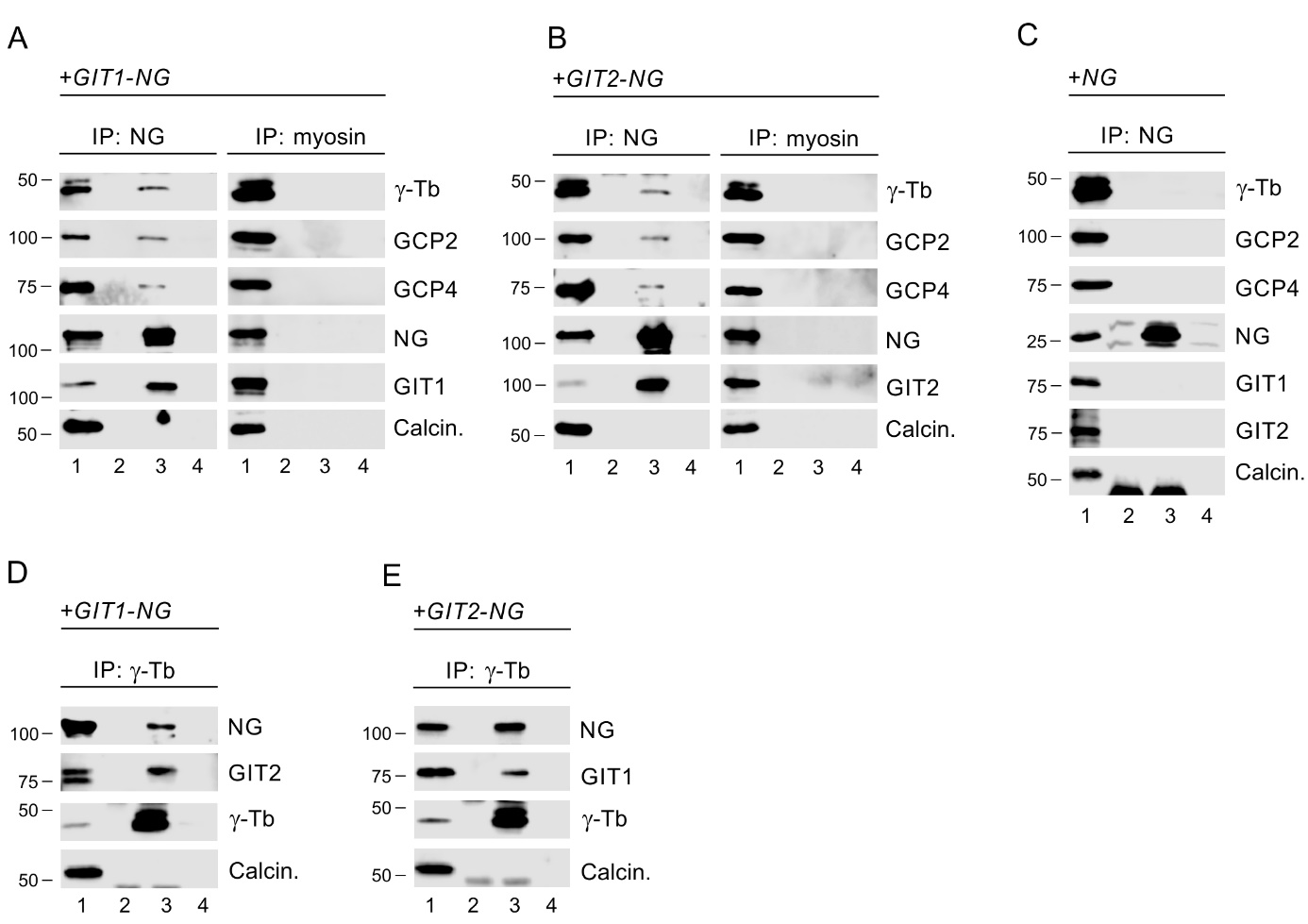


**Fig. S3** Exogenous GIT1 and GIT2 interact with γTuRC proteins. Immunoprecipitation experiments were performed using whole-cell extracts from U-251 MG cells expressing NG-tagged GIT1 (GIT1-NG), GIT2 (GIT2-NG), or NG alone. (**A-C**) Precipitation with immobilized rabbit Ab to NG or rabbit Ab to myosin (isotype control). (**D-E**) Precipitation with immobilized mouse mAb TU-31 (IgG2b) to γ-tubulin (γ-Tb). Blots were probed with Abs to γ-tubulin (γ-Tb), GCP2, GCP4, NG, GIT1, GIT2, and calcineurin (Calcin.; negative control). Load (*lane 1*), immobilized Abs without cell extracts (*lane 2*), precipitated proteins (*lane 3*), and Ab-free carriers incubated with cell extracts (*lane 4*).


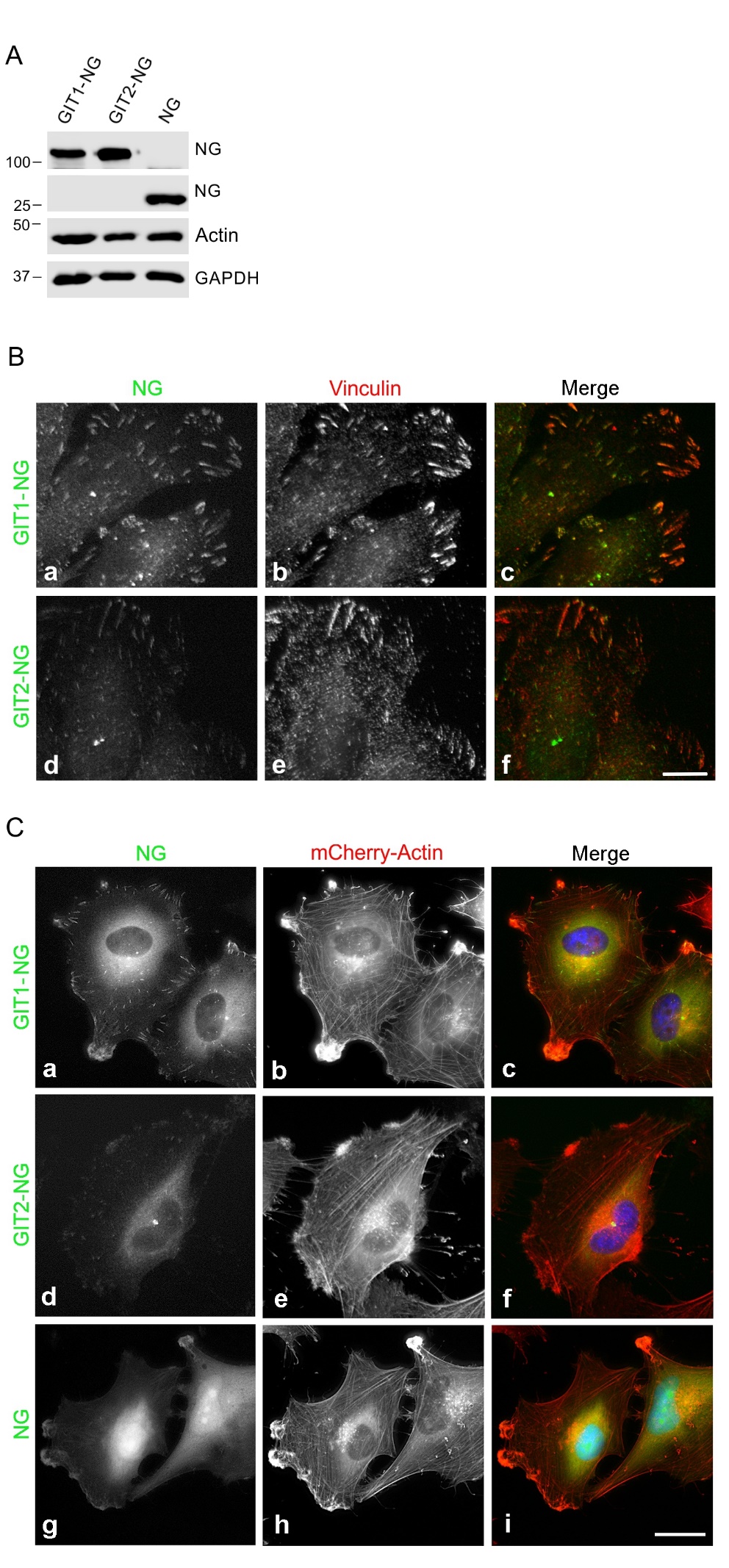


**Fig. S4** Expression and subcellular distribution of GIT1 and GIT2 in glioblastoma cells. U-251 MG cells (**A, B**) or U-373-MG expressing mCherry-actin (**C**) were transfected with plasmids for NG-tagged GIT1 (GIT1-NG), GIT2 (GIT2-NG), or NG alone. (**A**) Immunoblot analysis of whole-cell lysates. The blot was probed with Abs to NG, actin, and GAPDH. (**B**) Fluorescence staining of U-251 MG cells. GIT1-NG (**a**), vinculin (**b**), and superposition of images (**c**; GIT1-NG in green and vinculin in red). GIT2-NG (**d**), vinculin (**e**), and superposition of images (**f**; GIT2-NG in green and vinculin in red). The images (**a, d**; **b, e**) were collected and processed in the same manner. Fixation Tx/F/M. Scale bar for panels **a-f**, 10 μm. (**C**) Fluorescence staining of U373-MG cells. GIT1-NG (**a**), mCherry-actin (**b**), and superposition of images (**c**; GIT1-NG in green, mCherry-actin in red, DAPI, in blue). GIT2-NG (**d**), mCherry-actin (**e**), and superposition of images (**f**; GIT2-NG in green, mCherry-actin in red, DAPI in blue). NG (**g**), mCherry-actin (**h**), and superposition of images (**i**; NG in green, mCherry-actin in red, DAPI in blue). The images (**a, d, g; b, e, h**) were collected and processed in the same manner. Fixation F. Scale bar for **a-i**, 20 μm.


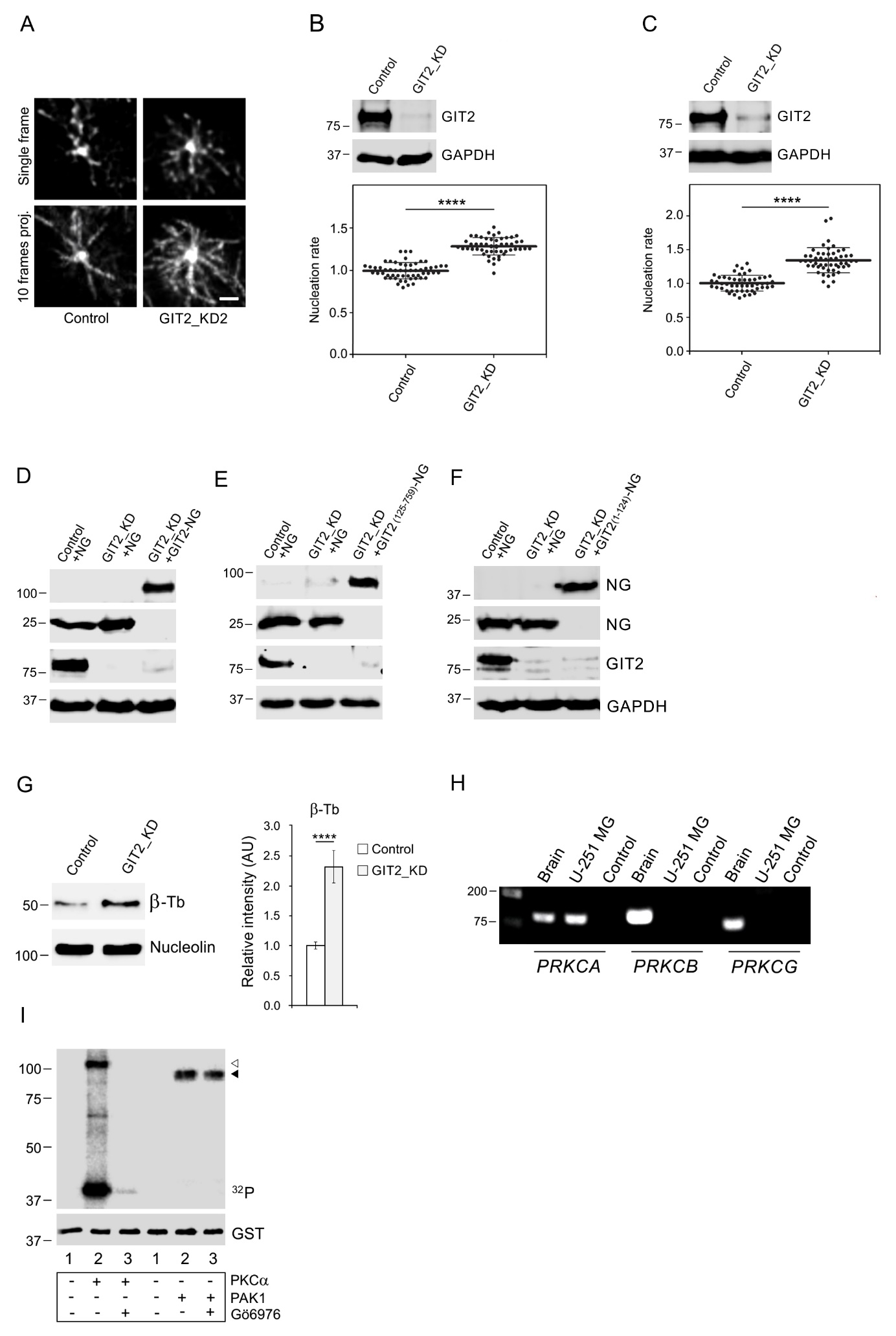


**Fig. S5** The effect of GIT2 depletion on microtubule nucleation and microtubule amount. Phosphorylation of GIT2 by PKCα. (**A**) Time-lapse imaging of U-251 MG control cells and cells with depleted GIT2 level. Still images of EB3 (single frame) and projection tracks of EB3 comets over 10 s (10 frames projection). Scale bar, 1 μm. (**B**) The effect of GIT2 depletion in U-87 MG cells on microtubule nucleation. At the top, a blot of whole-cell lysates probed with Abs to GIT2, and GAPDH (loading control). Below, comparison of microtubule nucleation rate (EB3 comets/min) in GIT2_KD cells relative to control cells. Three independent experiments were performed (at least 16 cells counted in each experiment): Control (pLKO.1-NT) (n = 57), GIT2_KD (n = 53). (**C**) The effect of GIT2 depletion in U-118 MG cells on microtubule nucleation. At the top, a blot of whole-cell lysates probed with Abs to GIT2 and GAPDH (loading control). Below, comparison of microtubule nucleation rate (EB3 comets/min) in GIT2_KD cells relative to control cells. Three independent experiments were conducted (at least 14 cells counted in each experiment): Control (pLKO.1-NT) (n = 51), GIT2_KD (n = 52). (**B-C**) The bold and thin lines within the dot plots represent mean ± SD. A two-tailed, unpaired Student’s *t*-test was performed to determine statistical significance. ****, p < 0.0001. **(D-F)** Immunoblot analysis of whole-cell lysates in phenotypic rescue experiments. Lysates from control U-251 MG cells expressing NG alone (Control+NG; **D-F**), GIT2_KD cells expressing NG alone (GIT2_KD+NG; **D-F**), and GIT2_KD cells rescued by GIT2-NG (GIT2_KD+GIT2-NG; **D**), GIT2_(125-759)_-NG (GIT2_KD+GIT2_(125-759)_-NG; **E**), and GIT2_(1-124)_-NG (GIT2_KD+GIT2_(1-124)_-NG; **F**). The blots were probed with Abs to NG and GIT2, with GAPDH serving as a loading control. (**G**) The effect of GIT2 depletion on microtubule amount. Control U-251-MG cells or cells with depleted level of GIT2 were extracted in 0.2% Triton X-100 at 37°C, and detergent-insoluble fractions were analyzed by immunoblotting with Abs to β-tubulin (β-Tb) and nucleolin (loading control). Densitometric quantification of immunoblots is shown on the right, displaying the relative intensity (AU) of β-tubulin normalized to control cells and to the amount of nucleolin. Values represent mean ± SD (n = 5). A two-tailed, unpaired Student’s *t*-test was performed to determine statistical significance. ****, p < 0.0001. (**H**) Expression profile of cPKC isoforms PKCα, PKCβ and PKCγ in U-251 MG cells. A gel-based RT-PCR analysis of PKCα (*PRKCA*), PKCβ (*PRKCB*), and PKCγ (*PRKCG*) is shown. Human brain served as a positive control, while samples without cDNA templates served as a negative controls. (**I**) PKCα, but not PAK1, phosphorylates the Arf-GAP domain of GIT2. GST-tagged Arf-GAP domain (GST-GIT2_(1-124)_) was immobilized on Glutathione-Sepharose beads and subjected to a kinase assay with active PKCα or PAK1 in the absence or presence of PKC inhibitor Gö6976 (10μM). Phosphorylated proteins were detected by autoradiography. GST-fusion protein without kinase (*lane 1*), with kinase (*lane 2*), and with kinase in the presence of Gö6976 (*lane 3*). White and black arrowheads indicates the positions of PKCα and PAK1, respectively.
